# Cu^2+^ Substitution modulates the structural and optical properties of MgAl hydrotalcites for photo-assisted Fenton-like degradation of ciprofloxacin

**DOI:** 10.1039/d6ra05478a

**Published:** 2026-09-14

**Authors:** Van Nhuong Vu, Thi To Loan Nguyen, Thi Thanh Huong Nguyen, Thi Mai Viet Ngo, Truong Xuan Vuong

**Affiliations:** a Faculty of Chemistry, Thai Nguyen University of Education No. 20 Luong Ngoc Quyen Street Thai Nguyen City 24000 Vietnam; b Faculty of Natural Sciences and Technology, TNU-University of Science Tan Thinh Ward Thai Nguyen City 24000 Vietnam xuanvt@tnus.edu.vn; c Applied Chemistry Research Group, Thai Nguyen University of Education No. 20 Luong Ngoc Quyen Street Thai Nguyen City 24000 Vietnam

## Abstract

Although Cu-containing layered double hydroxides (LDHs) have been widely investigated for light-assisted oxidation, the relationship between Cu substitution, crystallographic evolution, optical characteristics, and catalytic reactivity remains incompletely resolved. A pristine MgAl hydrotalcite (MgAlH) and Cu-substituted MgAl hydrotalcites (*n*-CuMgH; nominal Cu : Mg : Al ratios of 0 : 7 : 3–6 : 1 : 3) were synthesized by coprecipitation at a constant divalent-to-trivalent cation ratio. All compositions retained the layered framework, while increasing nominal Cu substitution modified their crystallographic, textural, and optical characteristics. From MgAlH to 5-CuMgH, the apparent band gap decreased to 1.86 eV, whereas the Urbach energy increased to 3.407 eV, corresponding to a pronounced change in the fitted optical absorption-edge response. Under H_2_O_2_-assisted visible-light irradiation, 5-CuMgH exhibited the highest CIP removal among the investigated compositions, reaching 95.5% after 210 min with an apparent pseudo-first-order rate constant (*k*_app_) of 0.032 min^−1^. Under dark H_2_O_2_-assisted conditions, CIP removal remained high at 89.9%, indicating that H_2_O_2_-assisted Fenton-like oxidation provides the major contribution, while visible irradiation affords additional kinetic enhancement. Scavenger experiments implicated ˙OH as an important apparent contributor, whereas AA and EDTA-2Na produced weaker inhibition; the limited selectivity of the scavengers precludes definitive assignment of individual ROS contributions. 5-CuMgH was the most active composition tested, although the investigated nominal composition range does not establish a unique Cu-content optimum. Overall, the concurrent evolution of crystallographic, optical, textural, and surface-chemical characteristics supports a multifactorial composition–property–reactivity relationship, without assigning causality to any single material descriptor.

## Abstract 2

This study elucidates how Cu substitution simultaneously tunes the crystallographic, optical, textural, and surface-chemical characteristics of MgAl-LDHs and their CIP degradation performance under H_2_O_2_-assisted visible-light irradiation.

## Graphical abstract

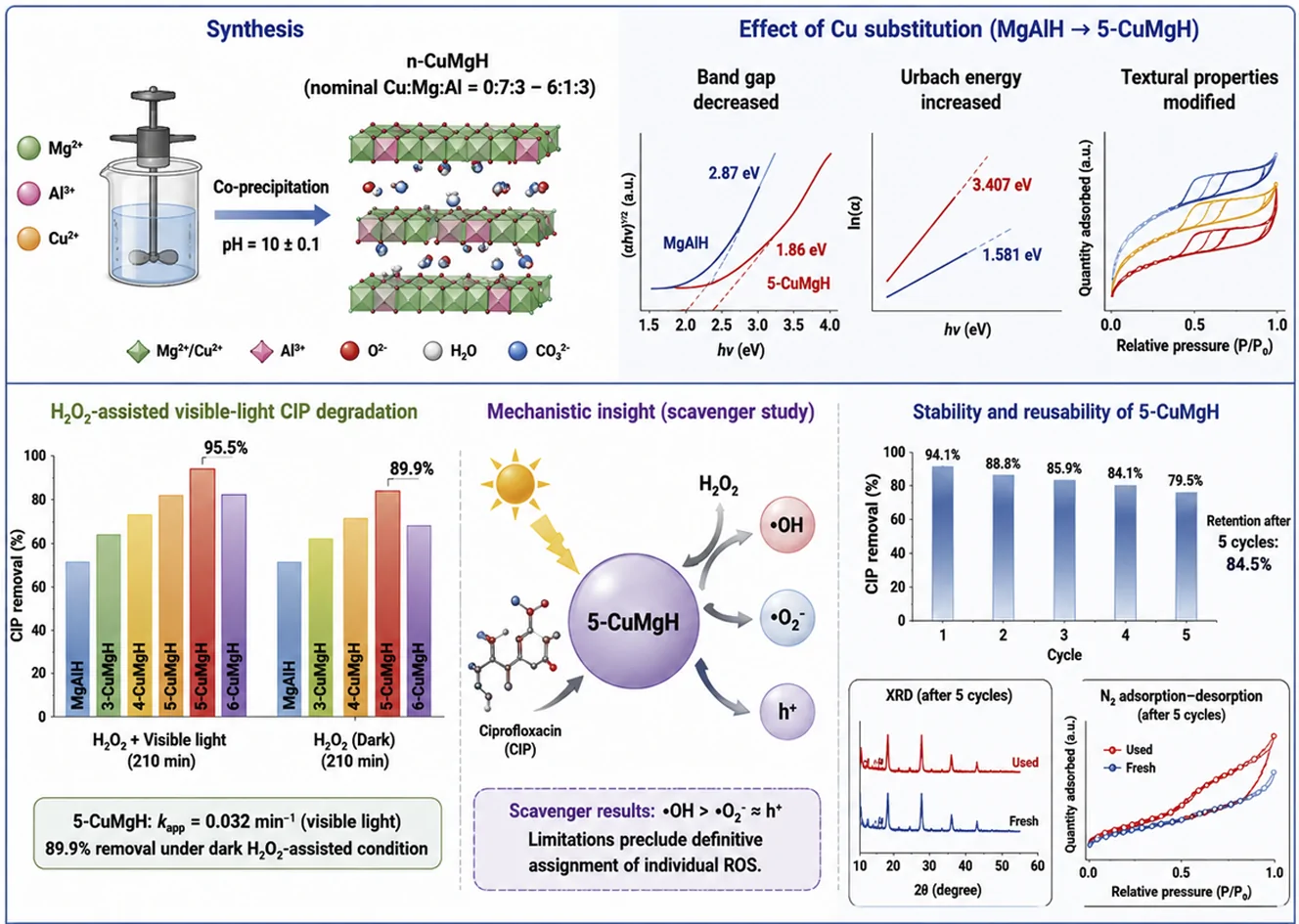

## 1. Introduction

The widespread use of antibiotics has significantly improved human and animal health since their introduction into clinical practice in the mid-twentieth century. Antibiotics are extensively employed not only in medicine but also in veterinary, aquaculture, and agricultural applications.^1–4^ However, the continuous increase in antibiotic consumption has resulted in their persistent release into aquatic and terrestrial environments. It has been estimated that approximately 30–90% of administered antibiotics are excreted unchanged or as active metabolites through urine and feces, eventually entering natural ecosystems.^2^

Numerous studies have reported the occurrence of antibiotic residues in surface water, groundwater, wastewater, sediments, and sludge at concentrations ranging from ng L^−1^ to mg L^−1^.^2,5–7^ For example, fluoroquinolone antibiotics have been detected in hospital wastewater at concentrations up to 16.8 µg L^−1^, while dozens of antibiotic compounds have been reported in reservoirs and river systems worldwide.^1^ Even at trace concentrations, these contaminants can exert adverse ecological impacts and promote the emergence and dissemination of antibiotic-resistant bacteria and antibiotic resistance genes, which have become major global environmental and public health concerns.^2,5,7^ Consequently, the development of efficient technologies capable of removing recalcitrant antibiotic contaminants from water systems remains an urgent challenge.

Various technologies have been investigated for antibiotic removal, including biological treatment, adsorption, ion exchange, membrane filtration, reverse osmosis, and chemical oxidation processes.^1,7^ Nevertheless, many conventional approaches suffer from limitations such as incomplete pollutant removal, membrane fouling, high operational costs, secondary pollution, and poor effectiveness toward persistent emerging contaminants.^7^ In contrast, advanced oxidation processes (AOPs) have attracted considerable attention because they can generate highly reactive oxygen species (ROS), including hydroxyl radicals (HO˙), sulfate radicals (SO_4_˙^−^), superoxide radicals (O_2_˙^−^), and singlet oxygen (^1^O_2_), which are capable of mineralizing complex organic pollutants into environmentally benign products.^1,2,7^ Among various AOPs, visible-light-driven photocatalysis is particularly attractive due to its environmental compatibility, low energy demand, operational simplicity, and potential utilization of solar energy.^8^

Layered double hydroxides (LDHs), also known as hydrotalcite-like compounds, have emerged as versatile functional materials for environmental remediation owing to their unique layered structure, tunable chemical composition, high anion-exchange capacity, memory effect, and structural flexibility.^9–11^ LDH-based materials have been extensively applied as adsorbents, catalysts, photocatalysts, drug carriers, and supports for environmental applications. In particular, pristine and modified LDHs, as well as mixed metal oxides derived from LDH calcination, have demonstrated promising performance for the removal and degradation of various contaminants, including heavy metals, dyes, and pharmaceutical compounds such as tetracycline, ciprofloxacin, sulfamethoxazole, doxycycline, ofloxacin, ibuprofen, and gemifloxacin.^12,13^

To further enhance photocatalytic activity, numerous transition metal ions, including Cu^2+^, Co^2+^, Fe^2+^/Fe^3+^, Ni^2+^, Mn^2+^, Cr^3+^, and Ti^4+^, have been incorporated into LDH structures.^10,12^ Among them, Cu-containing LDHs are particularly attractive because Cu species can participate in both photocatalytic and Fenton-like reactions, improve visible-light harvesting, facilitate charge transfer, and provide catalytically active sites.^10,12^ Consequently, various Cu-based LDH materials, including MgCuAl-LDH, CuZnAl-LDH, CuAl-LDH/polymer, CoCu-LDH, ZrCuFe-LDH/rGO, ZnCuFeCr-LDH, CuO/MgAl-LDH, Fe-Cu-LDH/Biochar, and Cu-Fe-LDH, have been developed for environmental remediation applications.^10,12^

Recent work in semiconductor photocatalysis has gradually shifted attention from catalytic composition alone toward the manner in which local structural environments shape electronic behavior.^14,15^ Crystalline imperfections, lattice distortion, and deviations from long-range order frequently alter the distribution of electronic states near the band edges, often with profound consequences for light absorption and charge transport.^14,15^ Among the available descriptors, Urbach energy (*E*_u_), obtained from the exponential absorption edge, has become a useful measure of electronic disorder because it reflects the extent of band-tail broadening and localized states in a solid.^16–18^ A broader Urbach tail is commonly accompanied by changes in the optical absorption edge and, in some systems, a reduction in the apparent band-gap energy.^16,17,19^ Whether such changes are beneficial depends strongly on the nature of the newly introduced states. They may facilitate visible-light utilization, yet excessive disorder can also promote charge-carrier recombination.

These considerations are well established for many conventional semiconductor photocatalysts, whereas their implications for LDH-based catalytic systems remain less clearly resolved.^20,21^ In particular, the extent to which Cu substitution in MgAl hydrotalcites alters crystallographic characteristics and optical band-tail behavior, and how these changes relate to catalytic response, remains insufficiently understood. Because variation in nominal Cu composition may coincide with changes in crystallographic ordering and optical absorption, while changes in surface chemical states may also contribute to catalytic response, changes in CIP removal cannot readily be assigned to a single material descriptor. Urbach-tail broadening is therefore considered here as an optical descriptor of band-tail behavior rather than as direct evidence of crystallographic disorder or atomistic electronic structure.

Previous studies on Cu-modified ZnAl-LDH and MgAl-LDH systems have shown that Cu-containing materials can alter catalytic behavior toward the degradation of dyes and industrial wastewater pollutants.^22–24^ More recently, Cu- and Co-containing hydrotalcites have also shown promising performance for CIP degradation.^25^ Yet, how variation in nominal Cu composition relates to crystallographic evolution, optical band-tail characteristics, and CIP-removal performance in Cu-substituted MgAl hydrotalcites remains insufficiently resolved. In addition, because H_2_O_2_ can promote CIP removal in the absence of irradiation, distinguishing oxidant-assisted dark oxidation from the additional enhancement produced by visible-light irradiation is essential for interpreting the observed reactivity.

Accordingly, this study examines the relationships among nominal Cu composition, crystallographic characteristics, optical band-edge characteristics, and CIP-removal performance in a series of Cu-substituted MgAl hydrotalcites (*n*-CuMgH) synthesized at a constant divalent-to-trivalent cation ratio. Particular emphasis is placed on the evolution of band-gap and Urbach-tail characteristics with nominal Cu composition and on distinguishing H_2_O_2_-assisted dark oxidation from the additional effect of visible irradiation. The *n*-CuMgH designations represent nominal precursor compositions rather than independently quantified bulk Cu concentrations. The materials were characterized by XRD, UV-vis DRS, XPS, and steady-state PL to examine crystallographic characteristics, optical properties, surface chemical states, and radiative recombination behavior, respectively. CIP-removal performance was evaluated under dark and visible-light conditions in the presence of H_2_O_2_. The study therefore evaluates experimentally supported relationships among nominal composition, structural characteristics, optical response, and CIP-removal performance. Mechanistic interpretations are treated as hypotheses requiring independent spectroscopic or theoretical verification.

## 2. Materials and methods

### 2.1. Chemicals and instrumentation

#### 2.1.1. Chemicals

Magnesium nitrate hexahydrate (Mg(NO_3_)_2_·6H_2_O), aluminum nitrate nonahydrate (Al(NO_3_)_3_·9H_2_O), copper nitrate trihydrate (Cu(NO_3_)_2_·3H_2_O), nitric acid (HNO_3_), and sodium carbonate (Na_2_CO_3_) were purchased from Sigma-Aldrich. Sodium hydroxide (NaOH, 96%) was obtained from Xilong (China). All chemicals were of analytical grade and were used without further purification. Ciprofloxacin (CIP) and hydrogen peroxide (H_2_O_2_, 30 wt%) were supplied by Sigma-Aldrich and were of analytical-grade purity. Ascorbic acid (AA), isopropyl alcohol (IPA), and ethylenediaminetetraacetic acid disodium salt (EDTA-2Na), used as reactive-species scavengers in the trapping experiments, were purchased from Xilong (China) and were of analytical-grade purity.

#### 2.1.2. Instrumentation

An IKA C-MAG HS 4 hotplate magnetic stirrer, a Memmert UNB 500 drying oven (Germany), an HQ411d pH mV^−1^ meter (HACH), and a VE-125-220 single-stage vacuum pump (Palma) were used for material synthesis, filtration, and drying. A 30 W Philips LED lamp with an emission maximum at 464 nm was used as the visible-light source. The lamp was positioned vertically above the reaction solution at a fixed distance of approximately 15 cm from the liquid surface. The experiments were conducted in 500 mL glass reactors containing 250 mL of reaction solution, corresponding to a solution depth of approximately 5 cm and a reactor diameter of approximately 8 cm. The reaction suspension was continuously stirred at 500 rpm. These irradiation and reaction conditions were kept constant throughout all light-assisted experiments to ensure consistent comparisons among the investigated catalysts. The incident light power density at the reaction surface was not measured using a calibrated radiometer and therefore could not be reliably reported in mW cm^−2^.

### 2.2. Synthesis of MgAl hydrotalcite and Cu^2+^-substituted MgAl hydrotalcites

The synthesis procedure was adapted from our previous work with minor modifications. Briefly, mixed aqueous nitrate solutions containing Mg^2+^, Al^3+^, and Cu^2+^ ions at predetermined molar ratios were prepared and continuously stirred. The pH of the suspension was maintained at 9.0 ± 0.2 using 2.0 M NaOH solution. Subsequently, 25 mL of 0.6 M Na_2_CO_3_ solution was added dropwise, and the resulting gel was further stirred for 60 min. The suspension was then aged at 70 °C for 24 h, filtered, thoroughly washed with deionized water until neutral pH was reached, and dried at 80 °C for 24 h. The dried solids were finally ground into fine powders. A schematic illustration of the synthesis procedure is presented in Fig. 1.

**Fig. 1:**
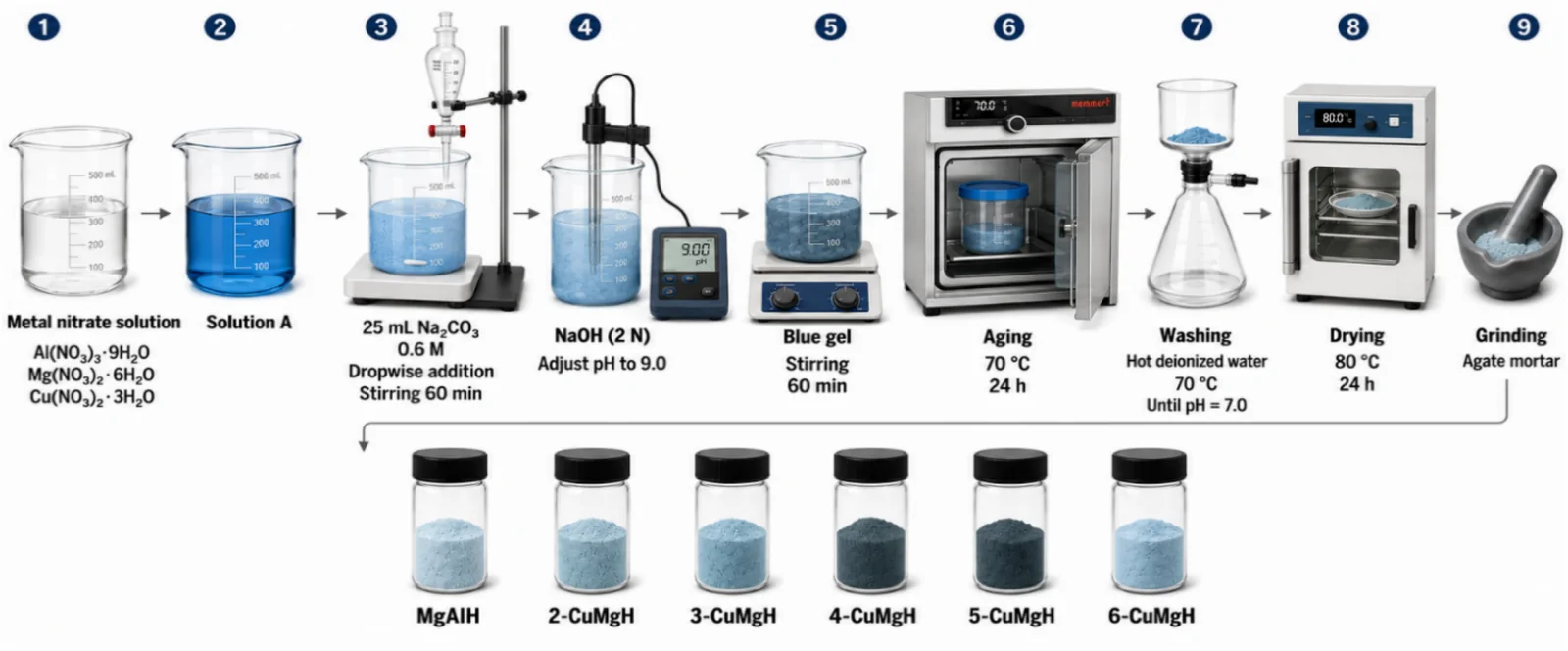
Schematic representation of the synthesis procedure for MgAl-LDH and Cu-substituted MgAl-LDH (CuMgH) samples.

The synthesized materials were designated as MgAlH (hydrotalcite), 2-CuMgH, 3-CuMgH, 4-CuMgH, 5-CuMgH, and 6-CuMgH (Cu^2+^-substituted MgAl hydrotalcites). Detailed molar ratios and precursor masses are provided in Table S1 of the (SI).

### 2.3. Structural and physicochemical characterization

The crystalline phases, layered structure, and crystallinity of the synthesized materials were characterized by X-ray diffraction (XRD) using a MiniFlex 600 diffractometer (Rigaku, Japan). The average crystallite size was estimated from the full width at half maximum (FWHM) of the characteristic diffraction peaks using the Scherrer equation. Variations in XRD peak broadening were also used to evaluate changes in structural ordering with increasing Cu^2+^ substitution.

Semi-quantitative elemental composition and elemental distribution were examined by energy-dispersive X-ray spectroscopy (EDS) using a HORIBA instrument (Japan). Surface morphology and platelet-like layered structures were examined by scanning electron microscopy (SEM, Hitachi S-4800, Japan).

The specific surface area (SBET), pore volume, and pore-size distribution were determined from N_2_ adsorption–desorption isotherms measured using a TriStar 3000 V6.07A analyzer (Micromeritics, USA). Prior to analysis, all samples were degassed under vacuum at 120 °C for 6 h to remove physically adsorbed moisture and volatile species.

X-ray photoelectron spectroscopy (XPS) was performed using a Kratos AXIS Ultra DLD spectrometer (Kratos Analytical, Shimadzu, Japan) equipped with a monochromatic Al Kα X-ray source (*hν* = 1486.6 eV). Survey spectra and high-resolution spectra of Cu 2p, Mg 1s, Al 2p, O 1s, and C 1s were acquired. The binding-energy scale was calibrated using the C 1s component at 284.8 eV. The Cu 2p spectra were analyzed to characterize the surface chemical state of Cu in the fresh material.

Steady-state photoluminescence (PL) spectra were recorded using FluoroMax Plus-C (Horiba, Japan) at an excitation wavelength of 350 nm over the emission range of 350–500 nm. The same instrumental parameters were used for all samples to enable comparative evaluation of the emission response.

Zeta-potential measurements were performed for MgAlH and 5-CuMgH over pH 4–10. For both materials, a constant suspension concentration of 1 g L^−1^ was used, and the suspension was equilibrated for 15 min after each pH adjustment before measurement. The pH was adjusted dropwise using 0.1 M HCl or 0.1 M NaOH, with the same equilibration period applied at each target pH. Zeta potential was measured using a Zetasizer Nano ZS90 instrument (Malvern Panalytical Ltd).

The optical properties of the materials were investigated by UV-vis diffuse reflectance spectroscopy (UV-vis DRS) in the wavelength range of 200–800 nm using a U-4100 spectrophotometer (Hitachi, Japan), with BaSO_4_ employed as the reflectance reference standard. The pseudo-absorption coefficient was obtained using the Kubelka–Munk transformation:

1 *F*(*R*) = (1 − *R*)^2^/2*R*

where *R* is the diffuse reflectance of the sample.

The optical band-gap energy (*E*_g_) was estimated using the Kubelka–Munk function combined with the Tauc approach according to:

2 (*F*(*R*)*hν*)^*n*^ = *A*(*hν* − *E*_g_)

where *hν* is the photon energy, *A* is a proportionality constant, and *n* = 1/2 was assumed for indirect electronic transitions in the investigated hydrotalcite materials. The *E*_g_ value was obtained by extrapolating the linear region of the Tauc plot to the photon-energy axis.

The Urbach energy (*E*_u_), which reflects the degree of electronic disorder and the formation of localized tail states near the band edges, was estimated from the exponential absorption-edge region using the Urbach relation. Because diffuse reflectance spectroscopy was employed, the Kubelka–Munk function *F*(*R*) was used as an approximation of the absorption coefficient for optical analysis.

3 *F*(*R*) = *F*_0_ exp(*hν*/*E*_u_)

The *E*_u_ value was obtained from the inverse slope of the linear region in the plot of ln[*F*(*R*)] *versus hν* according to:

4 *E*_u_ = [d(ln *F*(*R*))/d(*hν*)]^−1^

The zeta potential of the materials was measured using a Zetasizer Nano ZS90 instrument (Malvern Panalytical Ltd).

### 2.4. Evaluation of ciprofloxacin removal performance

#### 2.4.1. Calibration curve for ciprofloxacin determination

The concentration of ciprofloxacin (CIP) in aqueous solution was determined using an external calibration method. Standard CIP solutions with concentrations of 1.0, 2.0, 4.0, 6.0, 8.0, 10.0, 12.0, and 15.0 mg L^−1^ were prepared and analyzed by UV-vis spectrophotometry. Based on the measured absorbance values, a calibration curve was established according to the following linear relationship:

5 *y* = 0.0905*C*_CIP_ − 0.0021 (*R*^2^ = 0.9994)

where *y* represents the absorbance and *C*_CIP_ is the ciprofloxacin concentration (mg L^−1^). The calibration data and corresponding calibration curve are presented in Table S2 and Fig. S1 (SI).

#### 2.4.2. Adsorption experiments

The adsorption performance of the synthesized materials toward CIP was first evaluated under dark conditions. Adsorption experiments were carried out separately for each synthesized material. Briefly, 250 mL of a 25 mg L^−1^ CIP solution was transferred into six separate 500 mL glass reactors corresponding to MgAlH, 2-CuMgH, 3-CuMgH, 4-CuMgH, 5-CuMgH, and 6-CuMgH. To completely eliminate light interference, the reactors were wrapped with black plastic film and all light sources in the experimental room were switched off.

At predetermined time intervals of 30 min, aliquots (6–7 mL) were withdrawn, centrifuged, and diluted twofold prior to UV-vis analysis. The residual CIP concentration was then determined from the calibration curve. The adsorption equilibrium time and adsorption performance of each material were evaluated from the obtained concentration profiles.

The adsorption efficiency was calculated using eqn (6):

6 Adsorption efficiency (%) = ((*C*_0_ − *C*_*t*_)/*C*_0_) × 100

where *C*_0_ is the initial CIP concentration and *C*_*t*_ is the CIP concentration at time *t*.

#### 2.4.3. Photo-assisted Fenton-like degradation experiments

The reaction conditions were selected based on preliminary screening experiments to ensure reliable comparison among the synthesized materials. An initial CIP concentration of 25 mg L^−1^, a catalyst dosage of 0.2 g per 250 mL of solution, and 1.2 mL of 30 wt% H_2_O_2_ were employed throughout the catalyst-screening experiments.

To evaluate the contribution of each component in the reaction system, a series of control experiments was conducted under identical conditions, including: (i) CIP solution under LED irradiation without catalyst; (ii) CIP and catalyst in the dark; (iii) CIP and H_2_O_2_ without catalyst; (iv) CIP, catalyst, and H_2_O_2_ in the dark; (v) CIP and catalyst under LED irradiation without H_2_O_2_; and (vi) the complete photocatalytic system consisting of CIP, catalyst, H_2_O_2_, and LED irradiation. These experiments allowed the relative contributions of adsorption, direct photolysis, H_2_O_2_ oxidation, and photocatalytic degradation to be distinguished.

All experiments were independently performed at least three times, and the reported values represent the mean results. Experimental errors are expressed as standard deviations.

After adsorption equilibrium had been established in the dark for 30 min, 1.2 mL of 30 wt% H_2_O_2_ was added to each reaction mixture. The suspensions were subsequently irradiated using the 30 W LED lamp while being continuously stirred at ambient temperature. At 30 min intervals, 6–7 mL aliquots were collected, centrifuged, and diluted prior to concentration analysis.

The overall CIP removal efficiency was calculated using eqn (7):

7 Removal efficiency (%) = ((*C*_0_ − *C*_*t*_)/*C*_0_) × 100

where *C*_0_ and *C*_*t*_ represent the CIP concentrations at the initial stage and at reaction time *t*, respectively.

Because CIP adsorption onto the catalyst occurred during the dark equilibration period before irradiation, the degradation efficiency was evaluated relative to the CIP concentration remaining after adsorption equilibrium. After adsorption equilibrium was reached, H_2_O_2_ was added and the suspension was irradiated under visible light. The residual CIP concentration was monitored as a function of irradiation time, and the degradation efficiency was calculated according to eqn (8):

8 Photocatalytic degradation (%) = [(*C*_ads_ − *C*_*t*_)/*C*_ads_] × 100

where *C*_ads_ is the CIP concentration after adsorption equilibrium in the dark and *C*_*t*_ is the CIP concentration at irradiation time *t*.

After identifying the most active photocatalyst and the irradiation time required for near-complete CIP degradation, the effects of key operational parameters were further investigated. These parameters included solution pH, initial CIP concentration, the presence or absence of H_2_O_2_, and the presence or absence of visible-light irradiation. The experimental procedures followed those described above. Prior to UV-vis analysis, the pH of each sample was adjusted to its initial value to minimize spectral variations arising from pH-dependent changes in CIP speciation.

#### 2.4.4. Radical-trapping experiments

Reactive-species trapping experiments were performed to assess the participation of reactive species in the photocatalytic degradation of CIP. Isopropyl alcohol (IPA, 10 mM and concentrated IPA), ascorbic acid (AA, 1 mM), and ethylenediaminetetraacetic acid disodium salt (EDTA-2Na, 1 mM) were employed as scavengers for hydroxyl radicals (˙OH), superoxide radicals (˙O_2_^−^), and photogenerated holes (h^+^), respectively. The scavenger concentrations were selected based on previous studies.^25^

The experiments were carried out following the procedure described in Section 2.4.3. After the suspension had been stirred in the dark for 30 min and 1.2 mL of 30 wt% H_2_O_2_ had been introduced, 2.0 mL of the corresponding scavenger solution was added to the reaction mixture. The suspensions containing scavengers and the control suspension without scavengers were then irradiated simultaneously under identical conditions.

The scavenger concentrations were selected based on previously reported photocatalytic studies and were maintained constant throughout all trapping experiments to enable direct comparison of radical-quenching effects.

The inhibition extent observed in the presence of different scavengers was used to identify the dominant reactive species participating in CIP degradation and to support the proposed photocatalytic mechanism.

#### 2.4.5. Stability and reusability tests

The stability and reusability of 4-CuMgH were evaluated through consecutive photocatalytic cycles. Although 5-CuMgH was the most active composition tested in the catalytic screening experiments, insufficient 5-CuMgH remained after the preceding screening and characterization experiments for the repeated-cycle tests. Therefore, 4-CuMgH was selected for the recycling experiment. For the first cycle, six parallel reactors containing 250 mL of 25 mg L^−1^ CIP solution, 0.2 g of 4-CuMgH, and 1.2 mL of 30 wt% H_2_O_2_ were operated simultaneously under the optimized conditions.

Following 30 min of dark adsorption and 60 min of visible-light irradiation, approximately 7 mL of suspension was withdrawn from each reactor, centrifuged, diluted twofold, and analyzed for residual CIP concentration.

After completion of each cycle, the catalyst was recovered by filtration, repeatedly washed with hot deionized water (70 °C), and dried overnight at 70 °C before reuse in the subsequent cycle. The same procedure was repeated for successive cycles.

The variation in CIP removal efficiency over successive cycles was used to evaluate catalyst stability and the retention of photocatalytic activity during repeated operation.

### 2.5. Statistical analysis

All independent experiments were performed in triplicate unless otherwise stated, and results are reported as mean ± standard deviation (SD). For fitted parameters, including the apparent pseudo-first-order rate constant (*k*_app_), optical band-gap energy (*E*_g_), and Urbach energy (*E*_u_), 95% confidence intervals (95% CIs) were calculated from the replicate-level parameter estimates. Differences among the investigated compositions were evaluated by one-way analysis of variance (ANOVA), followed by Tukey's multiple-comparison test. Statistical significance was accepted at *p* < 0.05. All statistical analyses and graphical treatments were performed using Origin Pro 2019 (OriginLab Corporation, USA).

## 3. Results and discussion

### 3.1. Structural and physicochemical characterization of the synthesized materials

#### 3.1.1. XRD analysis of the synthesized materials

The X-ray diffraction (XRD) patterns of the synthesized MgAlH and Cu-substituted MgAl hydrotalcite samples (*n*-CuMgH) are presented in Fig. 2. The pristine MgAlH sample exhibited characteristic diffraction reflections at 2*θ* values of 11.40°, 23.10°, 34.75°, 38.93°, 46.27°, 60.91°, and 62.19°, corresponding to the (003), (006), (112), (115), (018), (110), and (113) crystallographic planes, respectively (PDF card No. 9009272). These reflections are characteristic of layered double hydroxides (LDHs) with a hydrotalcite-like structure and are consistent with previous reports on MgAl-based hydrotalcite materials.^2,26–28^

**Fig. 2:**
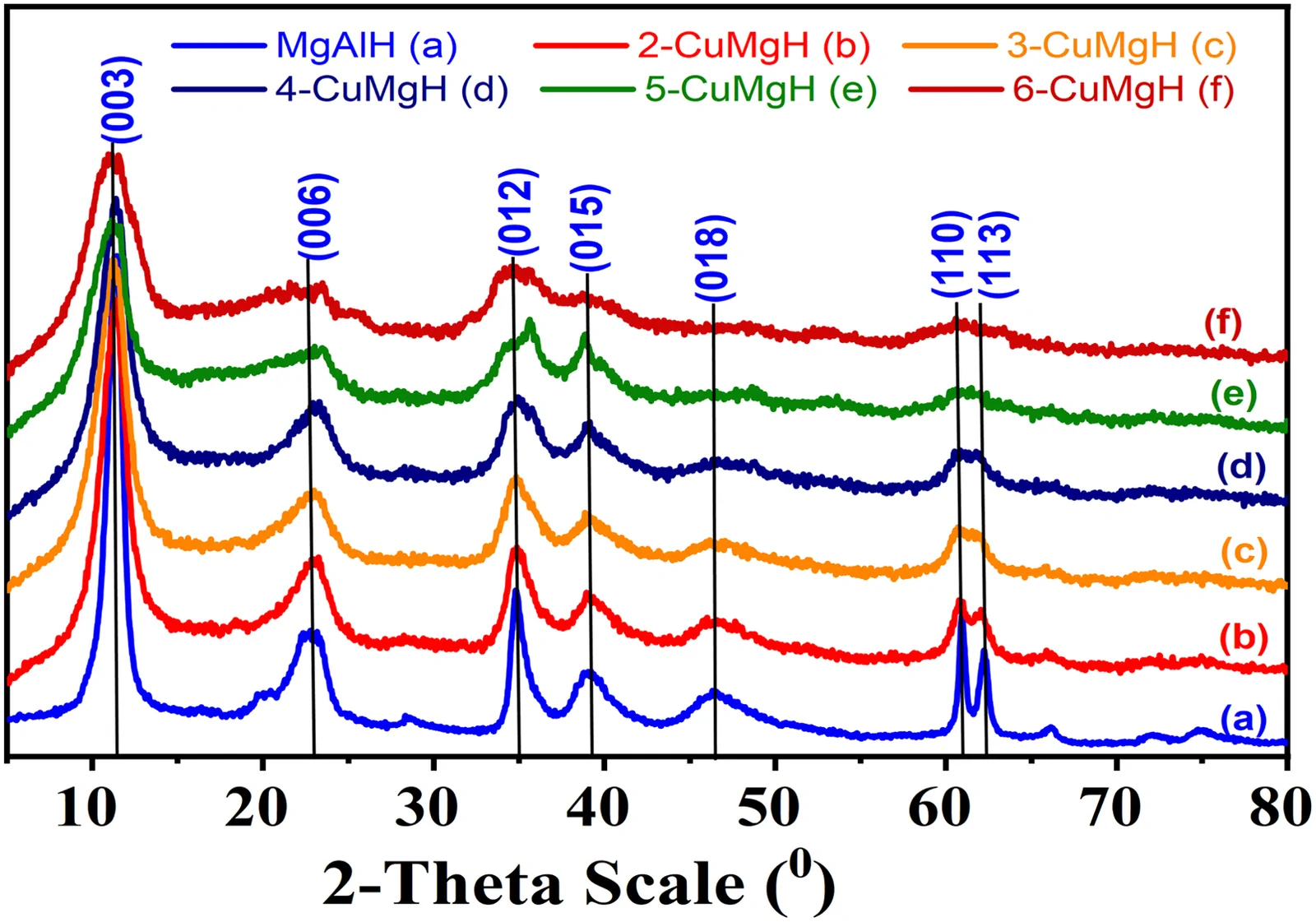
XRD patterns of pristine MgAlH and Cu-substituted MgAl hydrotalcites (*n*-CuMgH) with increasing Cu incorporation levels.

Following Cu^2+^ incorporation, all *n*-CuMgH samples retained the characteristic LDH reflections associated with the (003), (006), (012), (015), (018), (110), and (113) planes, indicating preservation of the layered hydrotalcite framework. However, a progressive decrease in diffraction peak intensity accompanied by noticeable peak broadening was observed as the Cu content increased. The broadening of the characteristic (003) reflection, reflected by an increase in its full width at half maximum (FWHM), indicates a reduction in coherent crystallite domain size and a gradual loss of crystallographic order within the layered framework. Such behavior is commonly associated with crystallite refinement together with changes in crystallographic coherence and/or increased lattice microstrain induced by cation substitution. However, XRD peak broadening alone does not uniquely quantify the degree of structural disorder and should therefore be interpreted independently of the electronic disorder discussed later through the Urbach energy analysis.^26,29^

In addition, the (006) reflection became progressively weaker and was no longer discernible for the 5-CuMgH and 6-CuMgH samples. The disappearance of this higher-order basal reflection suggests a substantial reduction in long-range stacking order along the *c*-axis direction of the LDH layers, further supporting the notion that increasing Cu incorporation perturbs the regular layered arrangement while preserving the overall hydrotalcite framework.

The basal reflections of all samples remained within a narrow range of 2*θ* = 11.2–11.4°, corresponding to *d*_003_ values between 7.72 and 7.89 Å (average 0.779 ± 0.01 nm). These interlayer spacings are characteristic of carbonate-intercalated hydrotalcite structures and indicate that CO_3_^2−^ remained the dominant interlayer anion throughout the synthesis process.^26^

The lattice parameters *a* and *c*, calculated according to *a* = 2*d*_110_ and *c* = 3*d*_003_, are summarized in Table 1. Parameter a represents the average cation–cation distance within the brucite-like layers, whereas parameter *c* corresponds to the periodic stacking distance along the crystallographic *c*-axis. Across the entire sample series, the lattice parameters remained relatively constant, with average values of *a* = 3.047 ± 0.01 Å and *c* = 23.358 ± 0.200 Å. The coexistence of nearly unchanged lattice parameters and progressively broadened diffraction peaks suggests that Cu incorporation appears to primarily affects local crystallographic coherence rather than inducing large-scale lattice expansion or collapse of the layered framework. Overall, the XRD results indicate progressive changes in crystallographic coherence and apparent crystallite dimensions with increasing nominal Cu composition, while the characteristic layered framework and interlayer spacing are largely retained.^30^

**Table 1:** XRD-derived structural parameters, lattice constants, and crystallite sizes of MgAlH and Cu-substituted MgAl hydrotalcites

Sample	*d*_003_ (Å)	*d*_006_ (Å)	*d*_110_ (Å)	*a* (Å)	*c* (Å)	Crystallite size (Å)
MgAlH	7.75	3.84	1.527	3.054	23.25	77.9
2-CuMgH	7.80	3.85	1.518	3.036	23.40	45.1
3-CuMgH	7.72	3.88	1.515	3.030	23.16	38.9
4-CuMgH	7.75	3.83	1.519	3.038	23.25	38.4
5-CuMgH	7.89	—	1.531	3.062	23.67	30.3
6-CuMgH	7.86	—	1.531	3.062	23.58	26.8

The average crystallite sizes were estimated using the Scherrer equation based on the full width at half maximum (FWHM) of the (003) reflection. As summarized in Table 1, the crystallite size decreased systematically from 77.9 Å for MgAlH to 45.1, 38.9, 38.4, 30.3, and 26.8 Å for 2-CuMgH, 3-CuMgH, 4-CuMgH, 5-CuMgH, and 6-CuMgH, respectively. This corresponds to an overall reduction of approximately 66% relative to the pristine MgAlH sample. The progressive decrease in crystallite size is fully consistent with the observed peak broadening and attenuation in the XRD patterns and suggests that increasing Cu incorporation hinders crystallite growth during LDH formation.

Because crystallite size estimated from the Scherrer equation does not distinguish size broadening from lattice microstrain, the present analysis is limited to a qualitative assessment of the crystallographic characteristics revealed by XRD. More rigorous separation of these contributions would require Williamson–Hall analysis or Rietveld refinement, which is beyond the scope of the present study.

Overall, the XRD results reveal that Cu incorporation progressively modifies several distinct crystallographic characteristics of the LDH framework, including crystallite refinement, attenuation of long-range stacking order, and changes in crystallographic coherence, while preserving the overall layered structure and interlayer spacing. These crystallographic characteristics provide the structural basis for interpreting the optical and photocatalytic behavior discussed in the following sections but should not be regarded as direct evidence of electronic disorder, which is evaluated independently through the Urbach energy analysis.

#### 3.1.2. SEM-EDS analysis of the synthesized materials

##### 3.1.2.1 SEM analysis

Representative SEM micrographs of MgAlH, 3-CuMgH, 5-CuMgH, and 6-CuMgH are presented in Fig. 3. These samples were selected to illustrate the morphological evolution accompanying progressive Cu addition during hydrotalcite synthesis. MgAlH represents the pristine LDH material, whereas 3-CuMgH, 5-CuMgH, and 6-CuMgH correspond to Cu-containing samples that exhibited progressively reduced crystallographic order according to the XRD analysis discussed above.

**Fig. 3:**
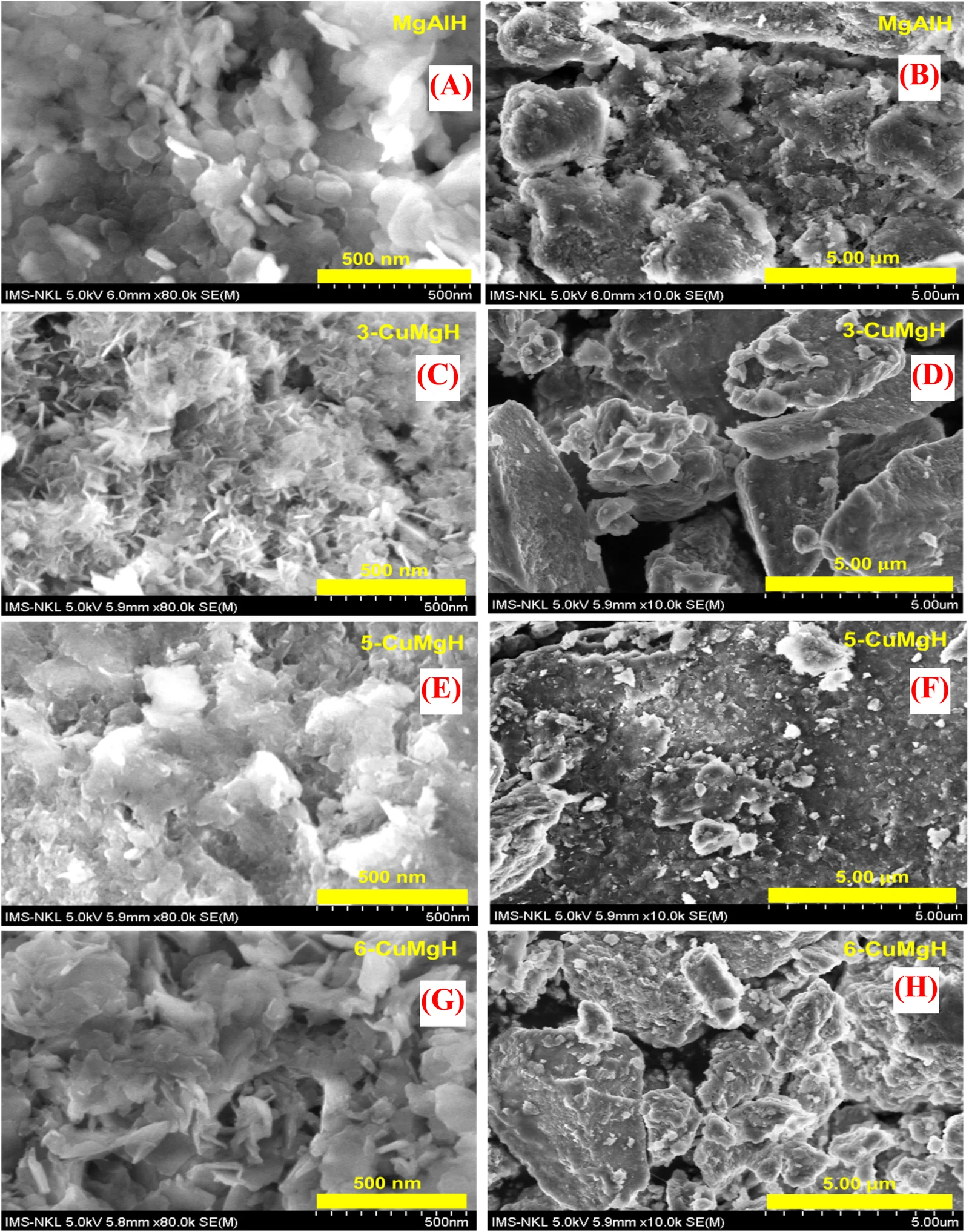
SEM micrographs of MgAlH (A and B), 3-CuMgH (C and D), 5-CuMgH (E and F), and 6-CuMgH (G and H) acquired at magnifications of 800 00× (A, C, E and G) and 100 00× (B, D, F and H). Scale bars correspond to 500 nm for the high-magnification images and 5 µm for the low-magnification images.

At high magnification (Fig. 3A, C, E and G), MgAlH displays the platelet-like morphology typically observed for hydrotalcite materials, consisting of relatively uniform lamellar crystallites arranged in loosely stacked aggregates. The lateral dimensions of these platelets are consistent with the comparatively larger crystallite size estimated from XRD analysis (Table 1). Following Cu addition, the layered morphology remained recognizable, although the platelets became less uniform and increasingly fragmented. Smaller lamellar domains were particularly abundant in the 5-CuMgH and 6-CuMgH samples, suggesting that higher Cu contents are associated with the formation of smaller and less well-defined layered domains.

##### 3.1.2.2 EDS analysis

EDS measurements were performed on MgAlH, 3-CuMgH, 5-CuMgH, and 6-CuMgH to examine the elemental composition of the synthesized materials (Fig. 4). The spectra contain signals corresponding to C, O, Mg, Al, and Cu, confirming the presence of the constituent elements in the synthesized materials.

**Fig. 4:**
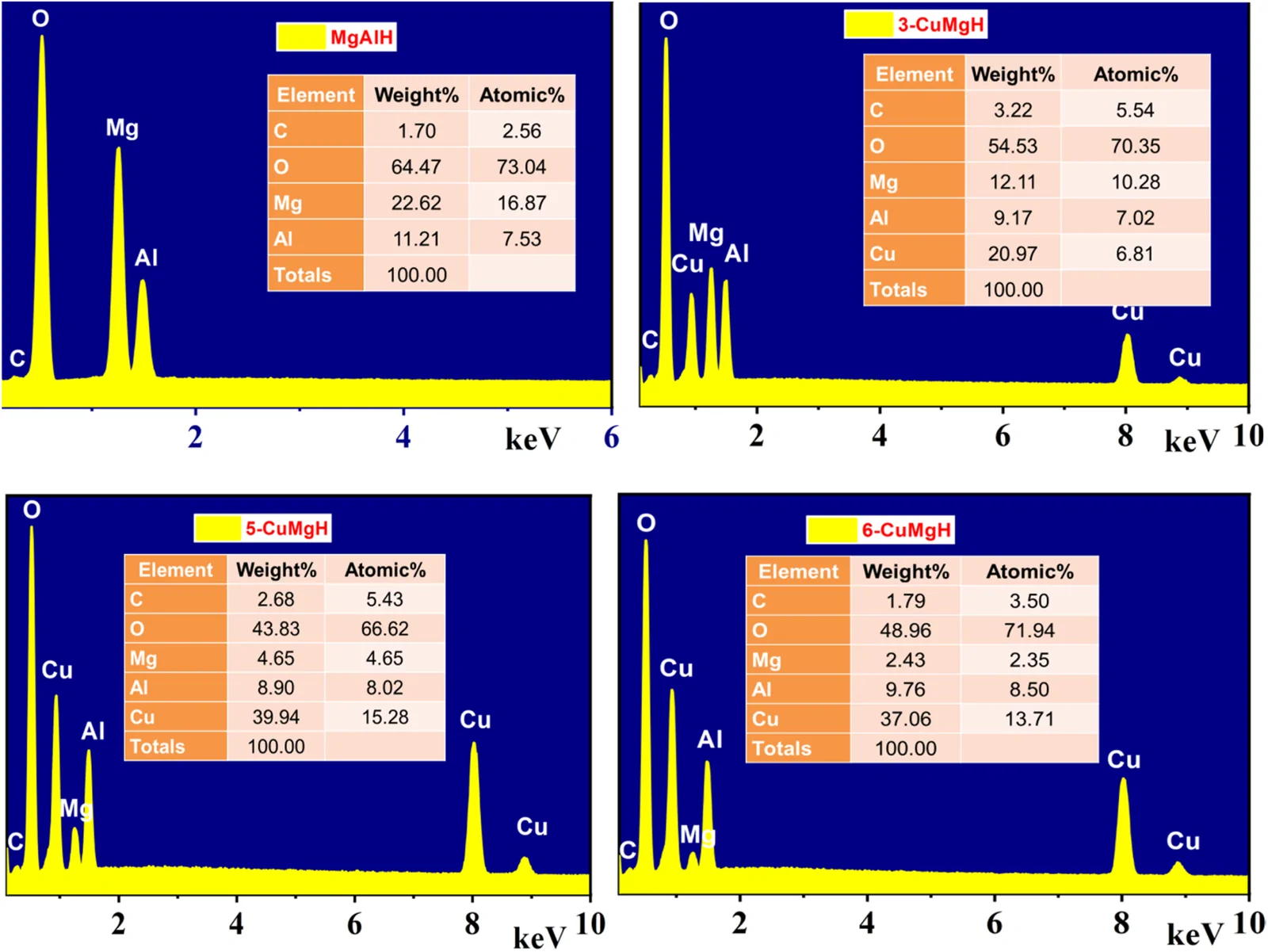
EDS spectra of MgAlH, 3-CuMgH, 5-CuMgH, and 6-CuMgH.

For MgAlH, the measured Mg : Al atomic ratio was generally consistent with the nominal synthesis composition, acknowledging the semi-quantitative nature of EDS. The Cu-containing samples likewise exhibited compositional trends that were consistent with the nominal precursor ratios, confirming the presence of Cu in the synthesized materials. The gradual increase in Cu-related EDS signals from 3-CuMgH to 6-CuMgH is consistent with the increasing nominal Cu composition used during synthesis.

Minor deviations between the measured and nominal elemental ratios were observed, particularly at higher Cu loadings. Such discrepancies are frequently encountered in coprecipitation-derived LDH systems and may arise from differences in precipitation behavior, local compositional variations, and the semi-quantitative nature of EDS analysis. Consequently, the EDS results should be interpreted as semi-quantitative compositional evidence supporting successful Cu incorporation rather than as definitive verification of the elemental stoichiometry.

When considered together with the XRD results, the SEM-EDS analyses suggest that increasing Cu content is accompanied by systematic changes in both elemental composition and morphology. The layered architecture characteristic of hydrotalcites remains preserved throughout the series, whereas smaller lamellar domains and reduced morphological uniformity become progressively more evident as the Cu content increases.

#### 3.1.3. N_2_ Adsorption–desorption isotherms of the synthesized materials

The N_2_ adsorption–desorption isotherms of the synthesized materials are presented in Fig. 5. All samples exhibit pronounced hysteresis loops over the relative pressure range of approximately *P*/*P*_0_ = 0.46–1.0, corresponding to type IV isotherms with H3 hysteresis according to the IUPAC classification. This adsorption behavior is characteristic of mesoporous structures associated with aggregates of plate-like particles and is consistent with the layered morphology observed by SEM.

**Fig. 5:**
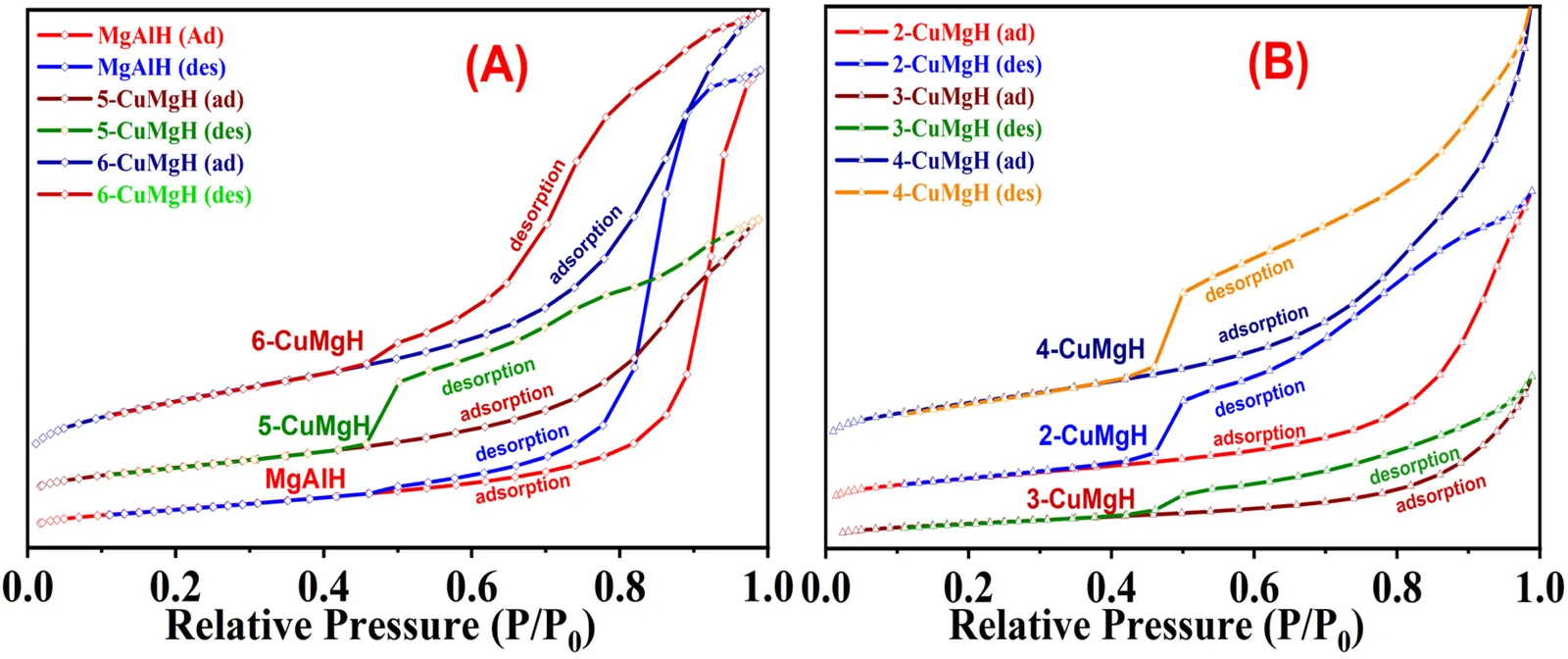
N_2_ Adsorption–desorption isotherms of MgAlH and Cu-substituted hydrotalcite materials (*n*-CuMgH): (A) MgAlH, 5-CuMgH, and 6-CuMgH; (B) 2-CuMgH, 3-CuMgH, and 4-CuMgH.

Although all samples display the same general isotherm type, notable differences are evident in the shape and extent of the hysteresis loops. The adsorption and desorption branches of MgAlH remain relatively close over the high-pressure region (*P*/*P*_0_ ≈ 0.8–1.0), whereas broader hysteresis loops are observed for the Cu-containing materials. This distinction is consistent with Cu-dependent changes in pore characteristics and the packing arrangement of the layered domains, as reflected by the combined N_2_ adsorption–desorption and SEM observations. Substantial differences are also apparent in the total amount of adsorbed nitrogen. MgAlH, 5-CuMgH, and 6-CuMgH exhibit considerably higher N_2_ uptake than 2-CuMgH, 3-CuMgH, and 4-CuMgH. The same tendency is reflected in the textural parameters summarized in Table 2 and Fig. 6.

**Table 2:** Textural properties of the synthesized materials derived from N_2_ adsorption–desorption measurements

No.	BET surface area (m^2^ g^−1^)	Total pore volume (cm^3^ g^−1^)	Average pore diameter (nm)
MgAlH	35.72	0.192	20.86
2-CuMgH	6.87	0.021	14.45
3-CuMgH	3.77	0.011	14.16
4-CuMgH	10.93	0.030	11.73
5-CuMgH	49.91	0.119	10.01
6-CuMgH	98.15	0.189	9.31

**Fig. 6:**
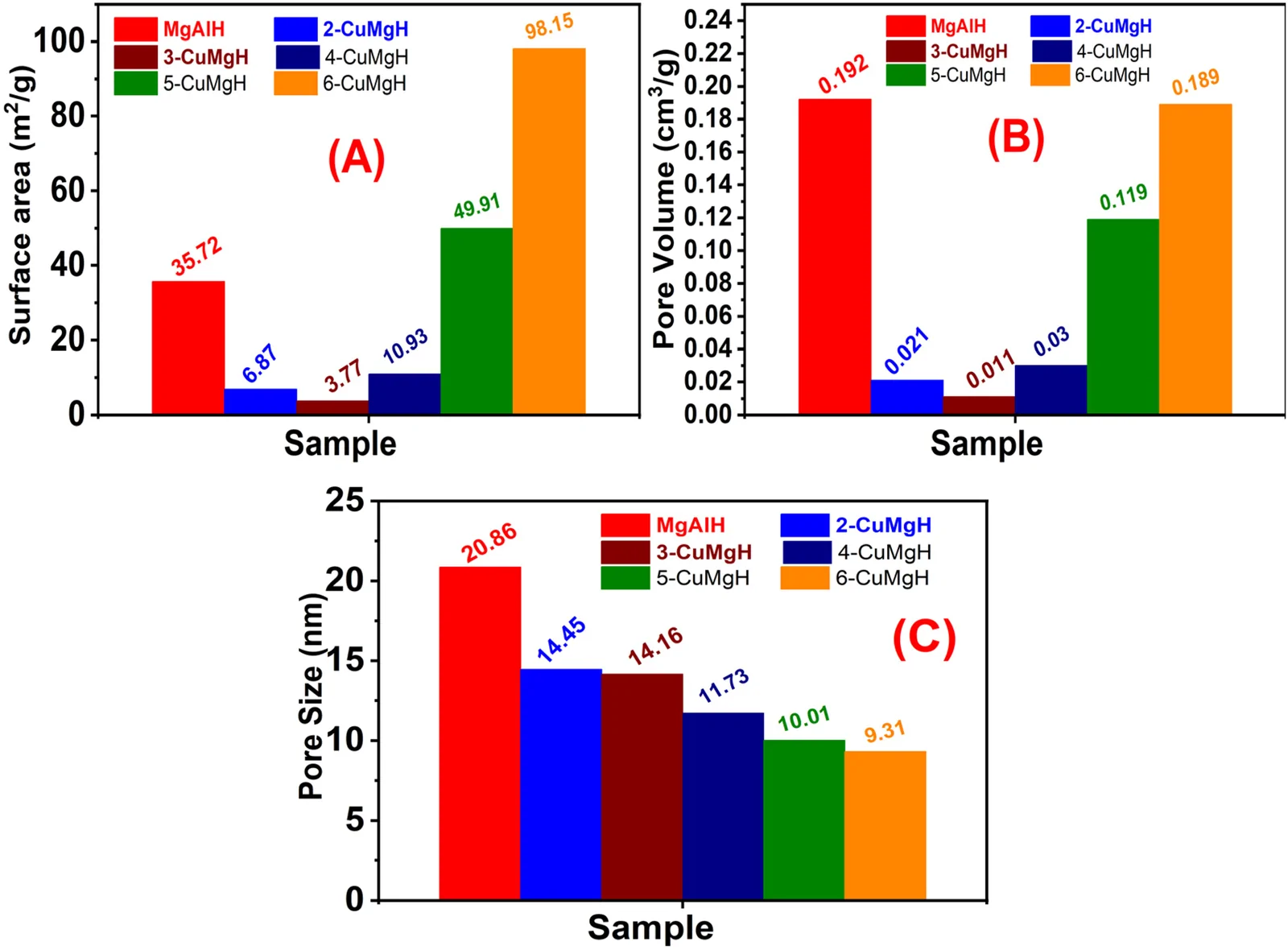
BET specific surface area (A), total pore volume (B), and average pore diameter (C) of the synthesized materials.

The BET surface area decreases sharply from 35.72 m^2^ g^−1^ for MgAlH to 6.87 and 3.77 m^2^ g^−1^ for 2-CuMgH and 3-CuMgH, respectively, before increasing to 10.93, 49.91, and 98.15 m^2^ g^−1^ for 4-CuMgH, 5-CuMgH, and 6-CuMgH, respectively. Similar variations are observed in the total pore volume.

The evolution of the textural properties is therefore distinctly non-monotonic. At low nominal Cu compositions, the BET surface area and pore volume initially decrease, reaching their lowest values for 3-CuMgH, and subsequently increase with further Cu incorporation. The pronounced increase observed for 5-CuMgH and 6-CuMgH indicates that the effect of Cu incorporation on textural development is composition-dependent rather than linear.

Interestingly, the marked increase in BET surface area observed for 5-CuMgH and 6-CuMgH coincides with a progressive reduction in apparent crystallite size and changes in crystallographic coherence revealed by XRD analysis. These concurrent changes are consistent with a combined influence of crystallite refinement, lamellar morphology, and particle/lamellar packing on the development of N_2_-accessible surface and interparticle void regions. Because the present XRD and SEM measurements characterize the final materials rather than the crystallization process itself, they do not establish a specific Cu-dependent nucleation or crystal-growth mechanism.

The average pore diameter decreases progressively from 20.86 nm for MgAlH to 9.31 nm for 6-CuMgH (Table 2). This decrease occurs concurrently with the morphological fragmentation observed by SEM, indicating that Cu incorporation is accompanied by systematic changes in the N_2_-accessible pore network. The progressive reduction in average pore diameter together with the increase in BET surface area at higher nominal Cu compositions suggests that the accessible textural network becomes increasingly dominated by smaller mesoporous and interparticle regions.

Overall, the XRD, SEM, and N_2_ adsorption results consistently demonstrate that Cu incorporation is accompanied by substantial changes in the crystallographic and textural characteristics of the LDH materials. While the characteristic layered hydrotalcite framework is retained, the materials exhibit smaller apparent crystallite dimensions, a progressively lower average pore diameter, and, at higher nominal Cu compositions, markedly increased BET surface areas. These experimentally observed structural and textural changes provide an important basis for interpreting the optical and catalytic behavior discussed in the following sections.

#### 3.1.4. UV-vis diffuse reflectance spectroscopy and optical band-edge characteristics

The UV-vis diffuse reflectance spectra of MgAlH and the Cu-modified hydrotalcite materials are presented in Fig. 7. Pristine MgAlH exhibits two absorption edges located in the ultraviolet region, approximately within 224–258 nm and 306–343 nm. These absorption features are commonly assigned to ligand-to-metal charge-transfer transitions involving oxygen and metal species within the hydrotalcite framework. Following Cu addition, all Cu-containing samples display enhanced absorption throughout the visible region, accompanied by a pronounced red shift of the absorption edge.

**Fig. 7:**
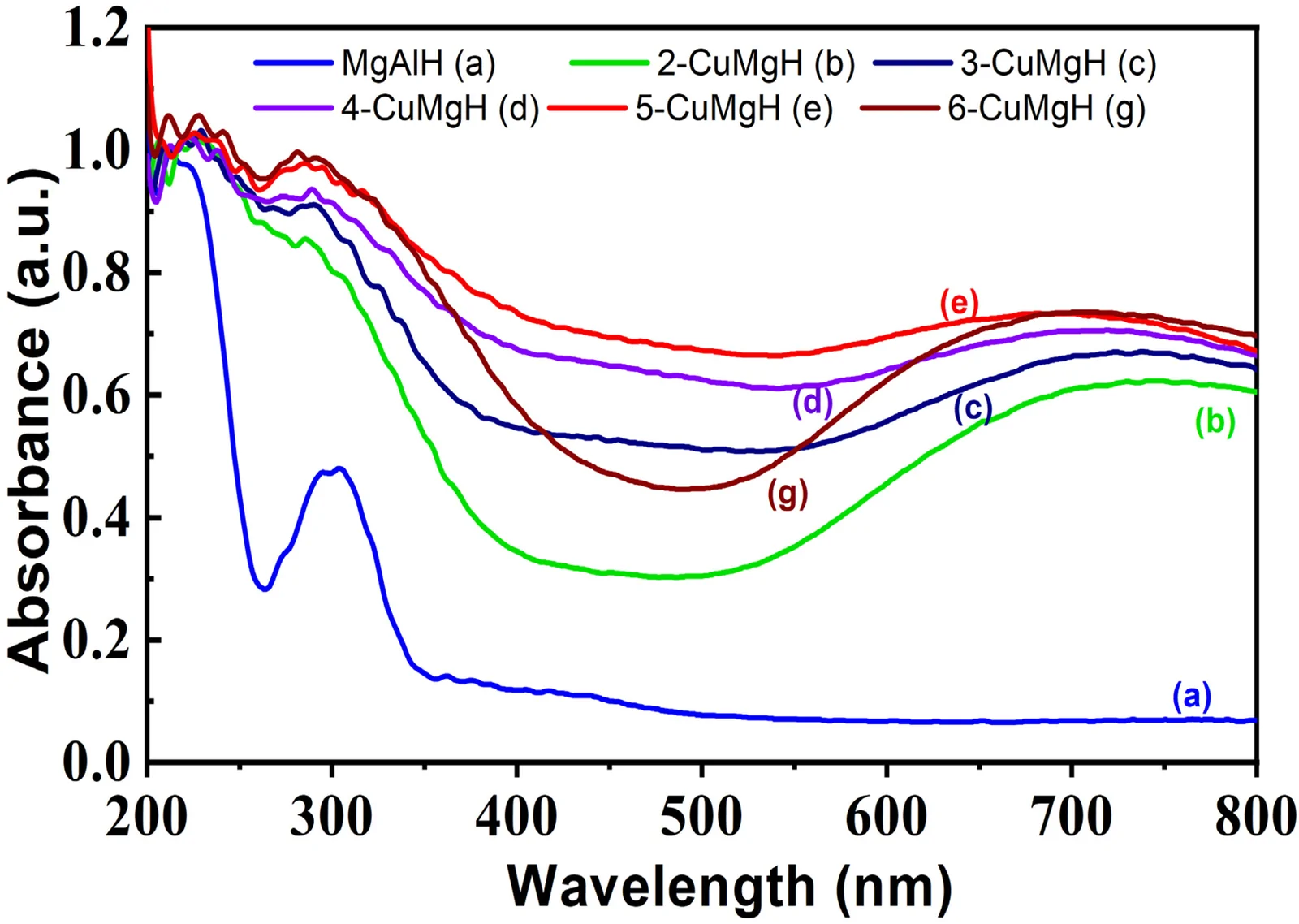
UV-vis diffuse reflectance spectra of MgAlH and Cu-modified MgAl hydrotalcite materials.

The extension of light absorption into the visible region is likely associated with additional electronic transitions introduced by Cu-containing species within the layered structure. From 2-CuMgH to 5-CuMgH, the absorption edge progressively shifts toward longer wavelengths as the Cu content increases. By contrast, 6-CuMgH exhibits a partial blue shift relative to 5-CuMgH, representing a deviation from the monotonic trend observed at lower Cu contents and suggesting a modification of the electronic environment at the highest level of Cu incorporation.

Band-gap energies (*E*_g_) were estimated using the Kubelka–Munk transformation coupled with Tauc analysis assuming an indirect electronic transition model (Fig. S2 (SI)). Urbach energies (*E*_u_) were derived from the exponential absorption-tail region to evaluate absorption-edge broadening within the synthesized materials (Fig. S3 (SI)). The corresponding values are summarized in Table 3. Representative Urbach plots together with the corresponding linear fitting regions are presented in Fig. S3 (SI). The selected fitting ranges exhibited excellent linearity, with correlation coefficients (*R*^2^) exceeding 0.99 for all materials, supporting the reliability of the calculated Urbach energies. The progressive broadening of the absorption-tail region observed for the Cu-containing samples is consistent with the increasing *E*_u_ values summarized in Table 3.

**Table 3:** Band-gap energies (*E*_g_) and Urbach energies (*E*_u_) of MgAlH and Cu-modified MgAl hydrotalcite materials^a^

Sample	Band gap, *E*_g_ (eV)	95% CI	Urbach energy, *E*_u_ (eV)	95% CI
MgAlH	3.55 ± 0.04^a^	3.451–3.649	0.347 ± 0.010^f^	0.322–0.372
2-CuMgH	2.68 ± 0.03^b^	2.593–2.767	0.983 ± 0.025^e^	0.921–1.045
3-CuMgH	2.52 ± 0.03^c^	2.433–2.607	1.758 ± 0.040^d^	1.659–1.857
4-CuMgH	2.06 ± 0.04^e^	1.973–2.147	3.142 ± 0.060^b^	2.993–3.291
5-CuMgH	1.86 ± 0.03^f^	1.773–1.947	3.407 ± 0.065^a^	3.246–3.569
6-CuMgH	2.42 ± 0.03^d^	2.333–2.507	1.416 ± 0.035^c^	1.329–1.503

a Values are presented as mean ± SD (*n* = 3). Different superscript lowercase letters within each column indicate significant differences among samples according to one-way ANOVA followed by Tukey's HSD test (*p* < 0.05).

Statistical analysis revealed highly significant composition-dependent differences in both optical parameters. One-way ANOVA showed significant differences among the six compositions for *E*_g_ (*p* = 9.53 × 10^−15^) and *E*_u_ (*p* = 1.83 × 10^−17^). Tukey's HSD test further identified significant pairwise differences among the investigated compositions, with different superscript letters indicating significant differences in Table 3. The corresponding 95% confidence intervals and detailed ANOVA and Tukey HSD results are provided in Table S3 (SI).

A pronounced decrease in apparent optical band-gap energy accompanies increasing nominal Cu composition from MgAlH to 5-CuMgH. The *E*_g_ value decreases from 3.55 eV for MgAlH to 2.68, 2.52, 2.06, and 1.86 eV for 2-CuMgH, 3-CuMgH, 4-CuMgH, and 5-CuMgH, respectively. For 6-CuMgH, *E*_g_ increases to 2.42 eV, consistent with the partial blue shift of its absorption edge relative to 5-CuMgH. Thus, the optical band-edge response exhibits a non-monotonic composition dependence, with the lowest apparent *E*_g_ observed for 5-CuMgH.

A non-monotonic trend is observed for *E*_u_. MgAlH exhibits the lowest *E*_u_ value (0.347 eV), whereas *E*_u_ increases across the series up to 5-CuMgH, reaching 3.407 eV, before decreasing to 1.416 eV for 6-CuMgH. The *E*_u_ values are treated here as empirical optical parameters describing the exponential absorption-tail behavior near the absorption edge.^16^ The value obtained for 5-CuMgH is unusually large and therefore warrants cautious interpretation. In diffuse-reflectance analysis, the extracted *E*_u_ may be sensitive to the selected fitting range and the assumptions associated with the Kubelka–Munk transformation and Urbach analysis.^31^ Thus, the particularly large *E*_u_ values obtained for 4-CuMgH and 5-CuMgH are associated with marked changes in the fitted optical absorption-edge response. Their absolute magnitudes should not, however, be interpreted as direct quantitative measures of microscopic band-tail width or as independent evidence for a specific defect, disorder mechanism, or electronic-state distribution.

The relationships among nominal Cu composition, *E*_g_, and *E*_u_ are summarized in Fig. 8. As shown in Fig. 8A, the apparent optical band gap decreases progressively from MgAlH to 5-CuMgH, followed by an increase for 6-CuMgH. Fig. 8B shows a corresponding increase in *E*_u_ from MgAlH to 5-CuMgH, followed by a decrease for 6-CuMgH. Thus, both optical parameters exhibit composition-dependent but non-monotonic behavior over the investigated range.

**Fig. 8:**
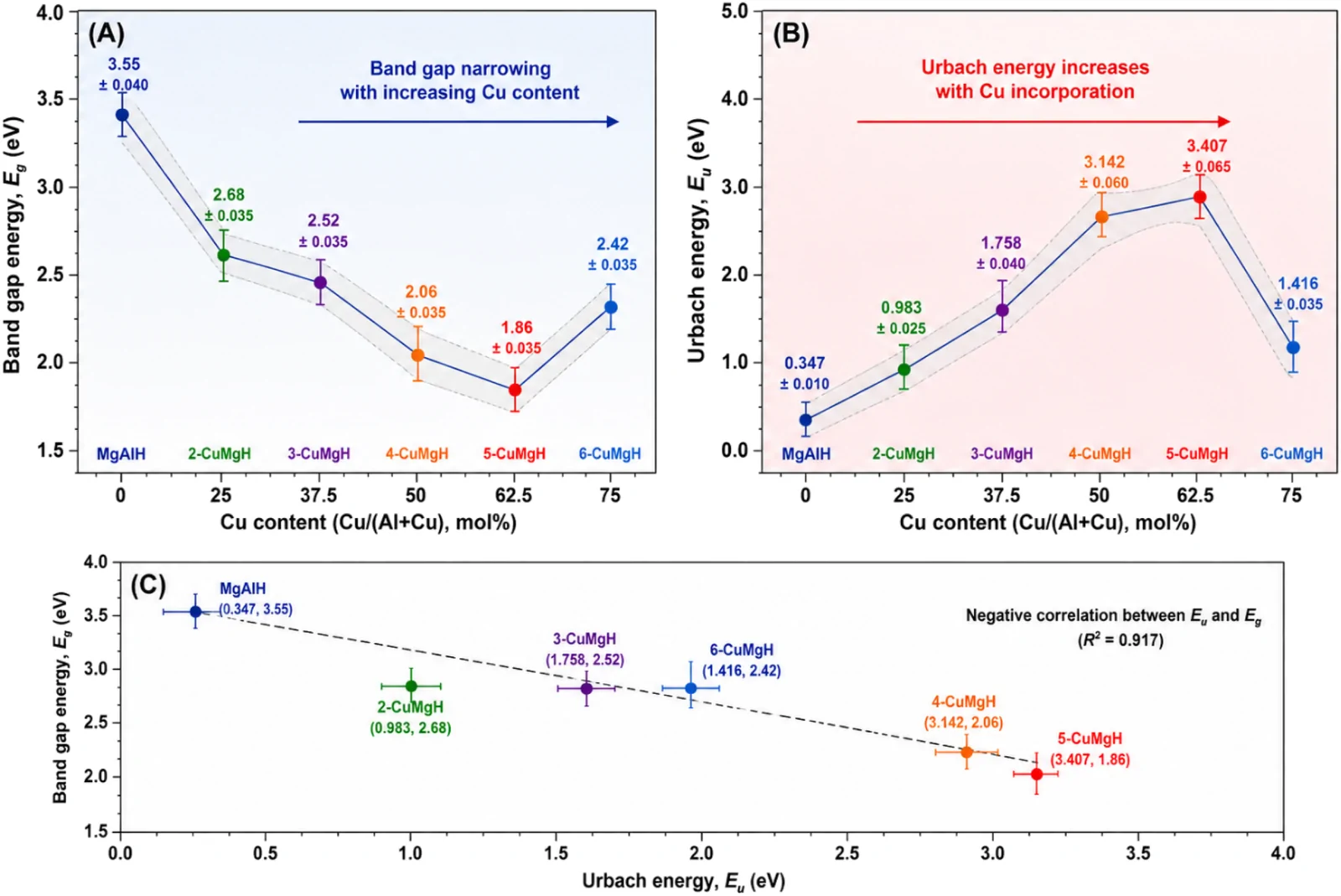
Evolution and interrelationship of the optical parameters of MgAlH and Cu-modified MgAl hydrotalcites as a function of Cu incorporation. (A) Dependence of the optical band-gap energy (*E*_g_) on Cu content. (B) Dependence of the Urbach energy (*E*_u_) on Cu content. Grey lines indicate the corresponding 95% confidence intervals. (C) Relationship between *E*_u_ and *E*_g_, showing an inverse linear correlation (*R*^2^ = 0.917).

Fig. 8C further examines the relationship between *E*_g_ and *E*_u_. Despite the limited number of compositions (*n* = 6), *E*_g_ and *E*_u_ exhibit an apparent inverse association over the investigated composition range (*R*^2^ = 0.917). This relationship is used descriptively rather than as evidence of a causal relationship between band-gap narrowing and Urbach-tail broadening. In particular, the deviation of 6-CuMgH from the trends observed for 2–5-CuMgH indicates that the optical response cannot be described by a simple monotonic dependence on nominal Cu composition.

When considered alongside the XRD results, the optical parameters and crystallographic characteristics show composition-dependent evolution. Samples exhibiting changes in diffraction characteristics and smaller apparent crystallite dimensions also show substantial changes in *E*_g_ and *E*_u_. However, these concurrent trends are treated as experimentally observed associations rather than evidence of a direct causal relationship between crystallographic coherence and Urbach-tail broadening. XRD and UV-vis DRS probe different aspects of the materials: XRD characterizes long-range crystallographic characteristics, whereas *E*_u_ describes the optical band-tail behavior near the absorption edge. Direct determination of atomistic electronic-state evolution would require complementary approaches such as DFT-based DOS/PDOS analysis or other electronic-structure-sensitive measurements, which were beyond the scope of the present study.

Among the synthesized materials, 5-CuMgH exhibits the lowest apparent optical band gap (1.86 eV) together with the highest Urbach energy (3.407 eV), indicating pronounced modification of its optical band-edge characteristics. In contrast, 6-CuMgH deviates from the trends observed for the other Cu-containing materials, exhibiting both a partial recovery of *E*_g_ and a decrease in *E*_u_. Collectively, the UV-vis DRS analysis demonstrates that nominal Cu composition substantially modifies the optical absorption and band-edge characteristics of the MgAl hydrotalcite materials, while the observed relationships with crystallographic characteristics remain experimentally supported associations rather than direct evidence of a common structural-disorder mechanism.

#### 3.1.5. XPS analysis

To further examine the surface chemical environment of the most active composition, X-ray photoelectron spectroscopy (XPS) was performed for the optimized 5-CuMgH sample. Because 5-CuMgH exhibited the highest visible-light-assisted Fenton-like degradation performance among the synthesized catalysts, it was selected for detailed surface chemical characterization. Owing to the surface-sensitive nature of XPS, the present analysis primarily probes the near-surface chemical states of the catalyst. Accordingly, the XPS results are interpreted alongside the XRD data, with XRD providing information on crystallographic characteristics and XPS providing information on elemental and chemical states.

The survey spectrum (Fig. 9a) confirms the presence of Cu, Mg, Al, O, and C at the catalyst surface, with no additional elements detected within the practical detection limits of the survey spectrum. The detection of Cu in the near-surface region is consistent with the Cu-containing nature of the synthesized material. Because XPS probes only the near-surface region, these data are not used to establish the bulk Cu : Mg : Al composition or the quantitative extent of Cu incorporation.

**Fig. 9:**
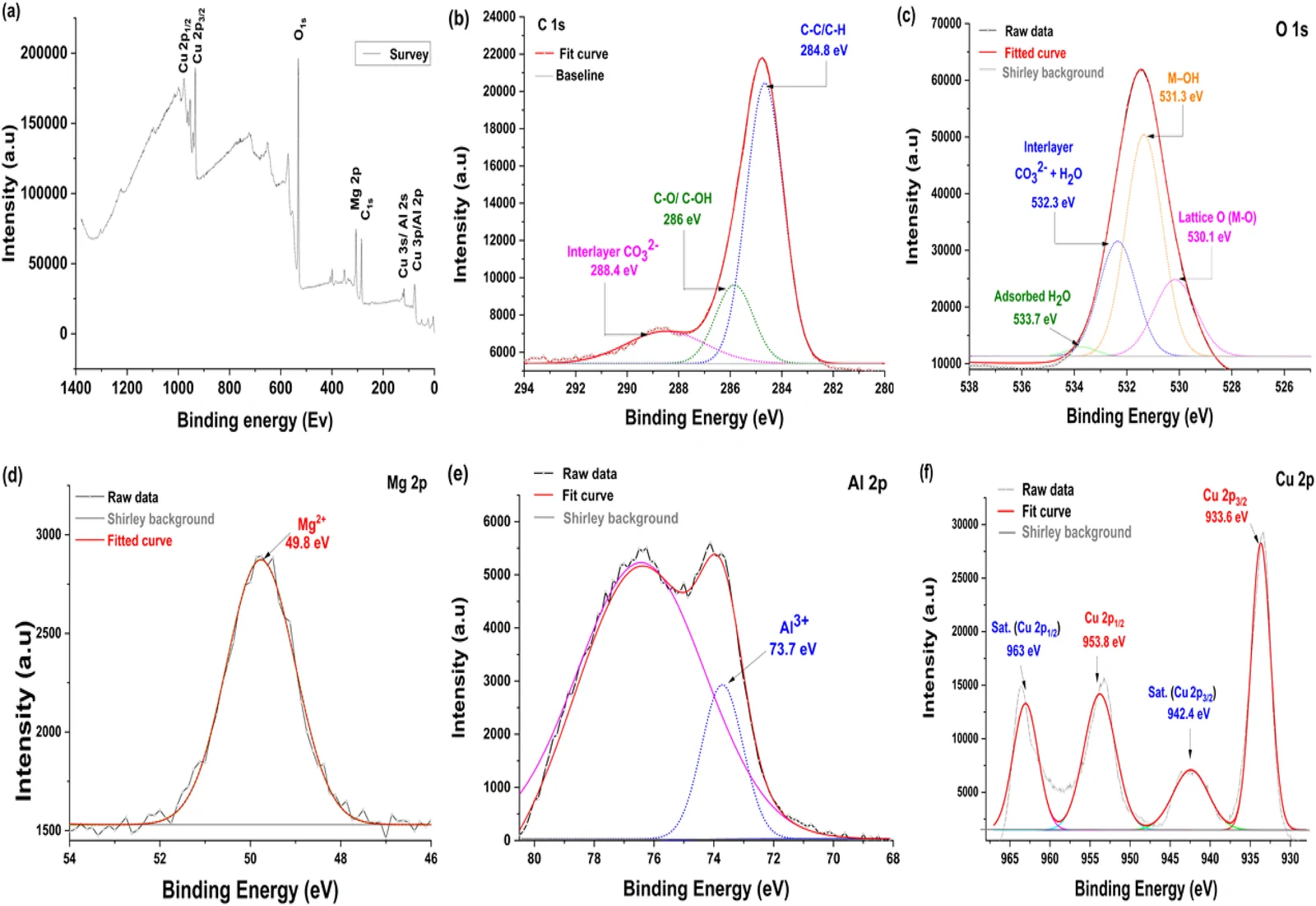
XPS characterization of Cu-MgAl-HLD: (a) survey spectrum, (b) C 1s, (c) O 1s, (d) Mg 2p, (e) Al 2p, and (f) Cu 2p high-resolution spectra.

High-resolution analysis of the C 1s region (Fig. 9b) reveals three components centered at 284.8, 286.0, and 288.4 eV, which are attributed to C–C/C=C, C–O/C–OH, and carbonate-related carbon species, respectively. The dominant C–C/C=C component at 284.8 eV was used as the binding-energy reference for spectral calibration. The component at 288.4 eV is consistent with carbonate-containing surface species, in agreement with the carbonate-containing LDH structure supported by the XRD results. Thus, the C 1s spectrum provides complementary information on the chemical environment associated with the carbonate-containing material.

In the O 1s region (Fig. 9c), four chemically distinguishable oxygen environments are resolved at 530.1, 531.5, 532.3, and 533.7 eV, consistent with lattice oxygen (M–O), structural hydroxyl groups (M–OH), carbonate-associated oxygen, and adsorbed water, respectively. These components are consistent with the characteristic surface chemistry of carbonate-containing layered double hydroxides. O 1s peak deconvolution alone does not provide unambiguous evidence for oxygen vacancies; accordingly, no oxygen-vacancy assignment or defect concentration is inferred from the present spectra. The analysis is restricted to the principal surface oxygen environments.

The Mg 2p and Al 2p spectra (Fig. 9d and e) exhibit characteristic binding energies of 49.8 and 73.7 eV, respectively, consistent with Mg^2+^ and Al^3+^ environments within the LDH framework. The preservation of the characteristic layered crystallographic structure is established primarily by XRD, whereas the XPS results provide complementary information on the corresponding chemical environment.

The Cu 2p spectrum (Fig. 9f) provides information on the initial chemical state of Cu in the fresh catalyst. The Cu 2p_3/2_ and Cu 2p_1/2_ photoelectron peaks located at 933.8 and 953.8 eV, respectively, are accompanied by pronounced shake-up satellite features centered at approximately 942.4 and 961.0 eV. The presence of these characteristic satellite features is consistent with Cu^2+^ being the predominant surface oxidation state in the as-prepared catalyst. A minor Cu^+^ fraction cannot be reliably quantified from the Cu 2p spectrum alone, and the absence of a clearly resolved Cu^+^ contribution should not be interpreted as evidence that Cu^+^ is completely absent. Reliable quantification of the Cu^+^/Cu^2+^ ratio would require complementary oxidation-state-sensitive measurements, such as Cu LMM Auger spectroscopy or X-ray absorption spectroscopy (XANES).

Importantly, XPS was performed only on the fresh catalyst prior to catalytic testing. The spectra therefore establish the initial surface chemical state of Cu but do not resolve its evolution during the degradation reaction. Comparative pre- and post-reaction XPS or other oxidation-state-sensitive measurements would be required to assess such changes. Accordingly, the present data do not provide direct evidence for a Cu^2+^/Cu^+^ redox cycle, and no explicit Cu redox cycle is inferred from the XPS results.

The XPS data indicate a Cu-containing surface dominated by Cu^2+^, together with Mg^2+^-, Al^3+^-, hydroxyl-, carbonate-, and adsorbed-water-related environments. Accordingly, in the mechanistic discussion, the XPS data are used to define the initial surface chemical state of Cu rather than to infer Cu oxidation-state cycling during catalysis.

#### 3.1.6. Photoluminescence characteristics

Photoluminescence (PL) spectroscopy was employed to examine the emission features and their intensity changes in MgAlH and 5-CuMgH. Both samples exhibit emission features centered at approximately 398, 436, and 468 nm, although their intensities differ after Cu incorporation (Fig. 10). The persistence of these emission regions in 5-CuMgH indicates that Cu incorporation modifies the observed emissive response rather than eliminating the overall emission features.

**Fig. 10:**
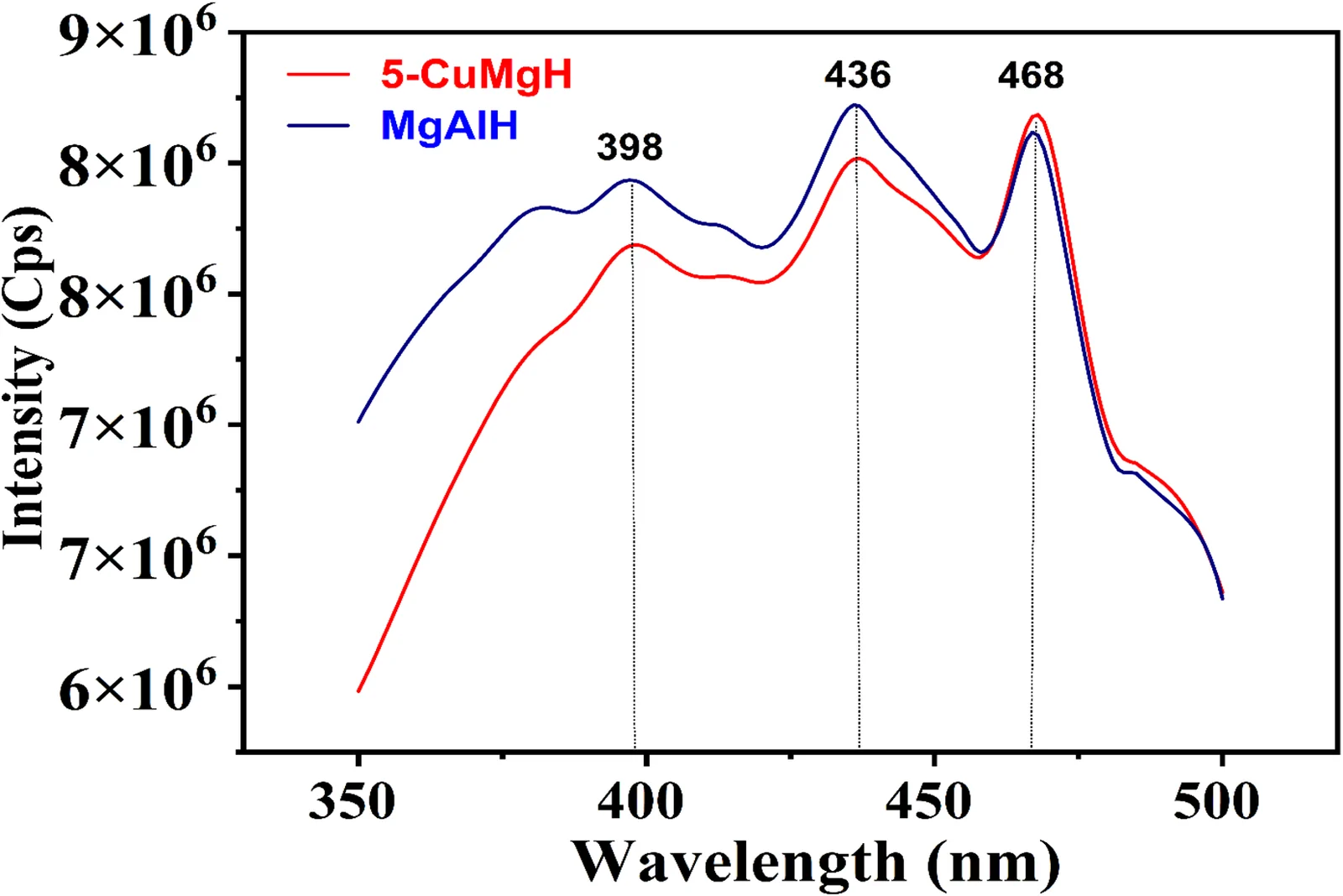
Photoluminescence spectra of MgAlH and 5-CuMgH. Cu incorporation modifies the relative emission intensities near 398, 436, and 468 nm, indicating changes in the PL response of the Cu-substituted hydrotalcite.

The most evident changes occur near 398 and 436 nm. Under identical measurement conditions, the emission intensity at approximately 398 nm decreases from 7.9 × 10^6^ cps for MgAlH to 7.5 × 10^6^ cps for 5-CuMgH, corresponding to a decrease of approximately 5%. Similarly, the emission intensity near 436 nm decreases from approximately 8.4 × 10^6^ to 8.0 × 10^6^ cps. These changes are consistent with a lower relative contribution of the corresponding emissive processes to the measured steady-state PL response following Cu incorporation. Because steady-state PL intensity is influenced by both radiative and non-radiative relaxation pathways, the observed attenuation cannot by itself distinguish whether it arises from changes in radiative recombination probability, non-radiative relaxation, emissive-state populations, or their combined effects.

In contrast, the emission near 468 nm remains essentially unchanged, with intensities of approximately 8.2 × 10^6^ and 8.3 × 10^6^ cps for MgAlH and 5-CuMgH, respectively. Cu incorporation therefore does not result in uniform PL attenuation across the investigated spectral range. Instead, the emission profile is altered while the ∼468 nm feature remains largely preserved. Because the spectra were not deconvoluted and no time-resolved PL measurements were performed, the individual emission features cannot be assigned unambiguously to specific Cu-related defects, trap states, or electronic transitions.

The PL profile changes after Cu incorporation, but the response is not consistent with simple global PL quenching. The attenuation of the ∼398 and ∼436 nm features is consistent with changes in their emissive contributions, whereas the nearly unchanged ∼468 nm feature indicates that its measured emission intensity is largely preserved under the present measurement conditions. These observations complement the XRD and UV-vis DRS results, which demonstrate concurrent changes in crystallographic characteristics, Urbach-tail broadening, and optical band-gap energy. These techniques probe different material properties and therefore should not be interpreted as independent direct evidence of a single microscopic electronic process.

For 5-CuMgH, the altered PL profile is consistent with changes in radiative and non-radiative relaxation under the tested conditions. Steady-state PL alone, however, cannot resolve these pathways or establish changes in charge-separation efficiency, electron–hole recombination kinetics, or carrier lifetime. Establishing such processes would require complementary measurements such as time-resolved PL, transient photocurrent, electrochemical impedance spectroscopy, or other charge-carrier-dynamics measurements.

Because PL measurements were performed only for MgAlH and 5-CuMgH, the present data do not establish how the emissive behavior evolves continuously across the complete Cu-composition series. Accordingly, the higher visible-light-assisted Fenton-like degradation performance of 5-CuMgH cannot be directly attributed to the observed PL changes. The PL response is therefore interpreted alongside the crystallographic, textural, optical, and surface-chemical characteristics of the materials.

### 3.2. Dark adsorption behavior of the synthesized materials toward ciprofloxacin

The dark adsorption behavior of the synthesized materials toward ciprofloxacin (CIP, 25 mg L^−1^) was evaluated before the subsequent photo-assisted Fenton-like degradation experiments. The adsorption profiles are presented in Fig. 11, and the corresponding numerical data are provided in Table S4 (SI).

**Fig. 11:**
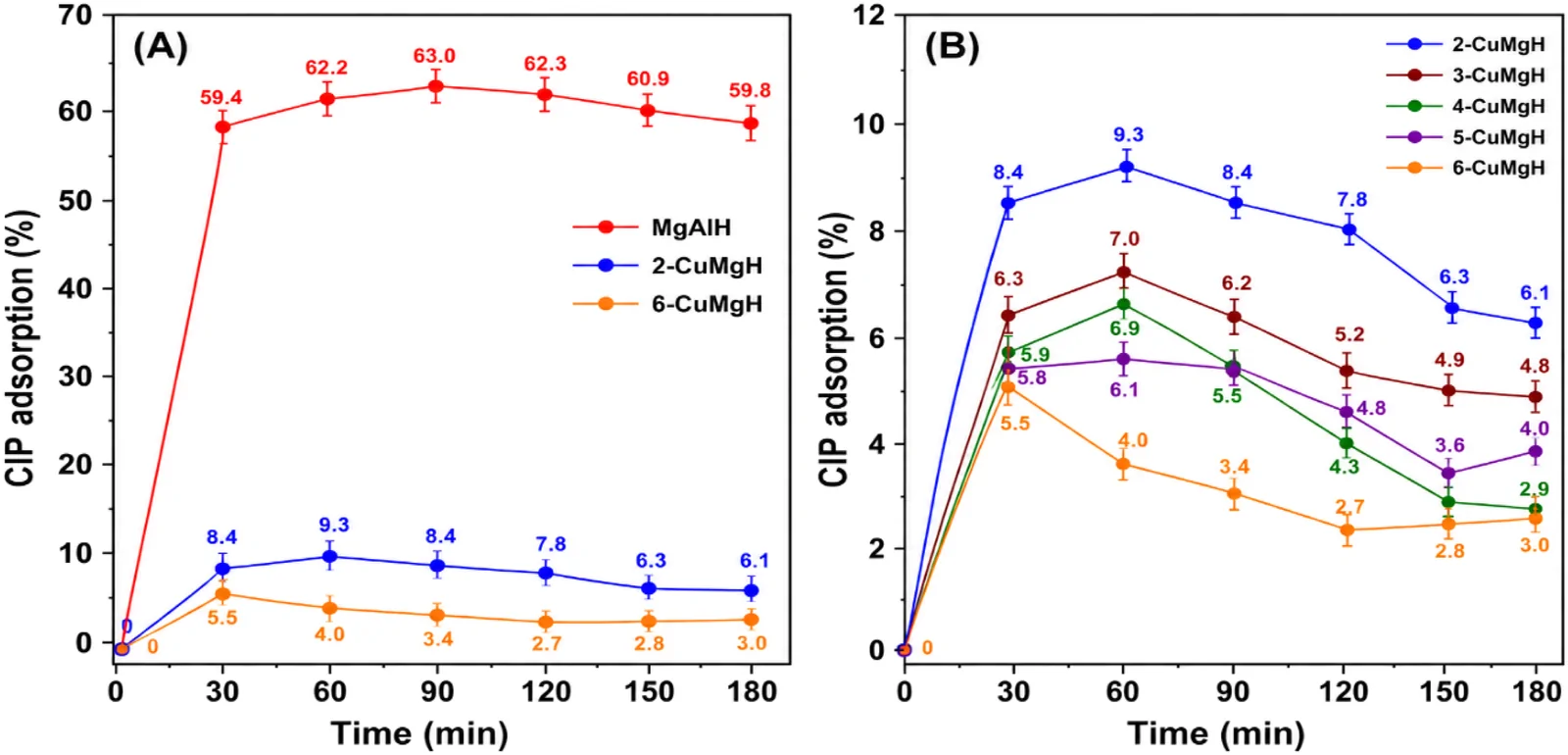
Dark adsorption of ciprofloxacin (CIP, 25 mg L^−1^) by MgAlH and Cu-containing MgAl hydrotalcites as a function of contact time. (A) Overall adsorption profiles. (B) Enlarged view of the Cu-containing samples. Error bars represent standard deviations (*n* = 3).

A pronounced difference in CIP uptake was observed between pristine MgAlH and the Cu-containing hydrotalcites (Fig. 11A). MgAlH showed rapid adsorption during the first 30 min, followed by a slower increase, reaching approximately 63.0% at 90 min. In contrast, all Cu-containing samples exhibited substantially lower uptake, remaining below 10% throughout the investigated period. Among the Cu-containing samples, 2-CuMgH showed the highest numerical adsorption, reaching 9.3% at 60 min, whereas 6-CuMgH exhibited the lowest uptake, remaining below 5.5%. The differences among the Cu-containing materials were relatively small compared with the pronounced contrast between MgAlH and the Cu-substituted materials.

Statistical analysis further supported the pronounced difference in CIP uptake at 30 min among the investigated materials. MgAlH exhibited an adsorption efficiency of 59.4 ± 0.78%, compared with 8.4 ± 0.46%, 6.3 ± 0.36%, 5.9 ± 0.36%, 5.8 ± 0.20%, and 5.5 ± 0.36% for 2-CuMgH, 3-CuMgH, 4-CuMgH, 5-CuMgH, and 6-CuMgH, respectively (Table S4, SI). One-way ANOVA revealed a significant difference among the six materials (*F*(5,12) = 6762.32, *p* = 2.92 × 10^−20^). Tukey's HSD test assigned MgAlH, 2-CuMgH, and the remaining four Cu-containing materials to three distinct statistical groups. MgAlH exhibited significantly higher adsorption than all Cu-containing materials, whereas 2-CuMgH was significantly higher than 3-CuMgH, 4-CuMgH, 5-CuMgH, and 6-CuMgH. No significant differences were detected among 3-CuMgH, 4-CuMgH, 5-CuMgH, and 6-CuMgH (*p* > 0.05). These results support the markedly lower CIP uptake of the Cu-containing materials at 30 min relative to pristine MgAlH under the standardized dark pre-contact condition.

Because most of the initial CIP uptake occurred within the first 30 min, this period was selected as the pre-contact time for the subsequent photo-assisted Fenton-like degradation experiments. This standardized condition limits the contribution of dark adsorption while providing a consistent initial condition for the light-assisted experiments. Importantly, 30 min does not represent complete adsorption equilibrium, particularly for MgAlH, for which CIP uptake continued to increase at longer contact times. Accordingly, the subsequent removal efficiencies were calculated relative to the CIP concentration remaining after the standardized 30 min pre-contact period.

The contrasting adsorption behavior cannot be explained by BET surface area alone. Although 5-CuMgH and 6-CuMgH possess substantially larger BET surface areas (49.91 and 98.15 m^2^ g^−1^, respectively) than MgAlH (35.72 m^2^ g^−1^), their CIP uptake is markedly lower. Conversely, 2-CuMgH exhibits the highest adsorption among the Cu-containing samples despite its relatively low BET surface area. These observations indicate that BET surface area is not the sole factor governing CIP uptake under the present experimental conditions.

To examine the possible contribution of surface electrostatics, pH-dependent zeta-potential measurements were performed for MgAlH and 5-CuMgH (Fig. S4, SI). Both materials remained positively charged over the investigated pH range of 4–10. The zeta potential of MgAlH decreased slightly from 35.4 mV at pH 4 to 33.7 mV at pH 10, whereas 5-CuMgH exhibited higher positive values, increasing from 45.1 mV at pH 4 to 48.8 mV at pH 6 before decreasing to 37.5 mV at pH 10. The difference between the two materials was most pronounced in the pH 6–8 range. Despite its higher positive zeta potential, 5-CuMgH exhibited substantially lower CIP uptake than MgAlH, indicating that surface electrostatics alone cannot account for the observed adsorption behavior.

CIP speciation was also considered based on reported p*K*_a_ values of approximately 6.1 and 8.7. CIP is an amphoteric fluoroquinolone whose predominant form changes from the cationic H_2_CIP^+^ species under acidic conditions to the zwitterionic HCIP ± form at intermediate pH and increasingly to the anionic CIP^−^ form under alkaline conditions (Fig. S5, SI). At the initial pH of approximately 6.5, the zwitterionic form is expected to predominate, although other protonation states may also be present. This speciation provides chemical context for interpreting possible electrostatic interactions, but the present measurements do not allow the contribution of individual CIP species to be quantified.

Together, the adsorption results suggest that CIP uptake reflects the combined effects of textural properties, surface electrostatics, CIP speciation, and surface-site characteristics rather than a single material descriptor. Because zeta-potential measurements were limited to MgAlH and 5-CuMgH, their implications are restricted to these two compositions and cannot be used to explain the adsorption behavior of the other Cu-containing samples, including 6-CuMgH.

Specific molecular interactions between CIP and the catalyst surfaces were not directly established in the present study. FTIR measurements of CIP-loaded catalysts were not performed; therefore, possible interactions involving surface functional groups or carboxylate–metal coordination cannot be established from the available evidence. Such interactions could be further examined using complementary post-adsorption characterization, including FTIR or other surface-sensitive spectroscopic techniques.

Overall, the Cu-containing materials exhibited limited dark adsorption under the investigated conditions, thereby providing a clearer basis for evaluating the additional CIP removal achieved under H_2_O_2_-assisted visible-light irradiation.

Adsorption and photocatalytic removal processes.

### 3.3. Ciprofloxacin removal performance of the synthesized hydrotalcite series

#### 3.3.1. Influence of irradiation time and nominal Cu substitution level on ciprofloxacin removal

Following the standardized 30 min dark pre-contact period described in Section 3.2, 1.2 mL of H_2_O_2_ (30 wt%) was added to the suspension, and CIP removal was monitored under irradiation from a 30 W LED source. The removal profiles are presented in Fig. 12A, and the corresponding kinetic and statistical data are summarized in Tables S5 and S6 (SI).

**Fig. 12:**
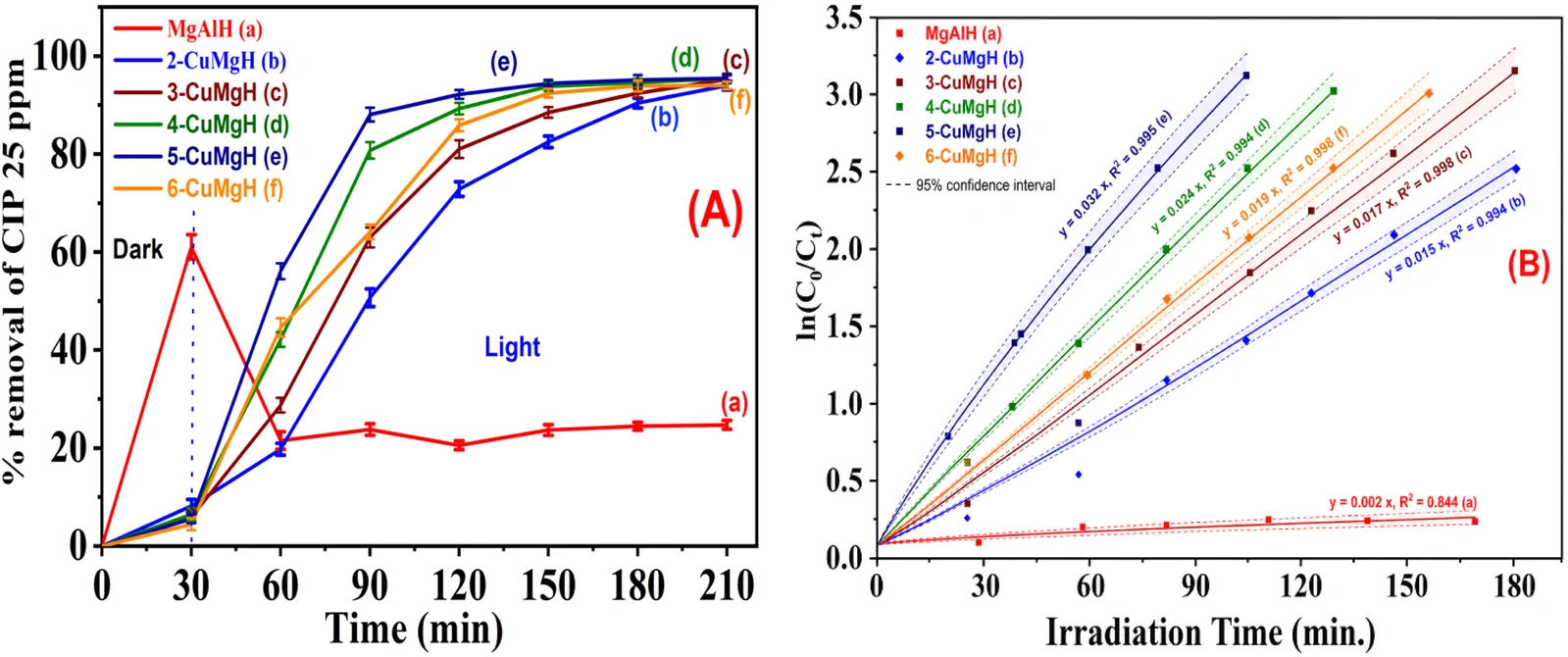
CIP removal profiles and pseudo-first-order kinetic analysis for MgAlH and Cu-substituted MgAl hydrotalcites under H_2_O_2_-assisted visible-light irradiation. (A) CIP removal as a function of reaction time (CIP, 25 mg L^−1^). (B) Pseudo-first-order kinetic plots.

The Cu-containing hydrotalcites exhibited distinct removal profiles. CIP removal increased rapidly during the first 90–120 min and subsequently approached a plateau. After 120 min, removal efficiencies were 72.8 ± 1.5%, 81.0 ± 1.8%, 89.3 ± 0.9%, 92.2 ± 0.9%, and 85.9 ± 1.2% for 2-CuMgH, 3-CuMgH, 4-CuMgH, 5-CuMgH, and 6-CuMgH, respectively. Thus, 5-CuMgH was the best-performing material within the investigated series, followed by 4-CuMgH, whereas further Cu substitution resulted in lower removal for 6-CuMgH.

MgAlH exhibited a markedly different concentration profile. Approximately 61.1% of CIP was removed during the 30 min dark pre-contact period, consistent with its substantially higher adsorption capacity. Following H_2_O_2_ addition and irradiation, the apparent cumulative removal decreased from 61.1% to 21.5%, indicating substantial desorption and/or redistribution of previously adsorbed CIP. Only limited net removal occurred thereafter, with the apparent removal remaining below 25% after 210 min. The MgAlH profile was therefore strongly influenced by adsorption–desorption processes and is not directly comparable with the Cu-containing materials, for which dark adsorption was comparatively minor.

Within the Cu-containing series, removal increased from 2-CuMgH to 5-CuMgH and then decreased for 6-CuMgH. This non-monotonic behavior shows that higher nominal Cu substitution does not necessarily improve CIP removal. The trend coincided with a decrease in apparent band-gap energy from 3.55 eV for MgAlH to 1.86 eV for 5-CuMgH and with enhanced visible-light absorption. However, the lower removal achieved by 6-CuMgH indicates that band-gap narrowing or visible-light absorption alone cannot account for the activity trend.

Pseudo-first-order analysis (Fig. 12B) provided a quantitative assessment of the apparent removal kinetics. The Cu-containing materials were well described by the model, with *R*^2^ values of 0.993–0.998. The corresponding *k*_app_ values were 0.015, 0.017, 0.024, 0.032, and 0.019 min^−1^ for 2-CuMgH, 3-CuMgH, 4-CuMgH, 5-CuMgH, and 6-CuMgH, respectively. MgAlH gave a poorer fit (*R*^2^ = 0.844) and an apparent *k*_app_ of 0.002 min^−1^. Because its concentration profile was strongly affected by adsorption–desorption, this fitted *k*_app_ should not be interpreted as a directly comparable degradation-rate constant. The kinetic parameters, 95% confidence intervals, and one-way ANOVA/Tukey HSD results are provided in Table S6 (SI). These statistical analyses support significant differences among the Cu-containing compositions but do not establish their mechanistic origin.

The compositional changes were accompanied by differences in crystallographic characteristics, optical absorption, Urbach-tail behavior, surface chemical states, and steady-state PL response. XPS identified Cu^2+^ as the predominant initial surface oxidation state of fresh 5-CuMgH, while PL revealed a modified emission profile relative to MgAlH. Neither measurement, however, provides direct evidence for Cu^2+^/Cu^+^ redox cycling or enhanced charge separation during reaction. Band-gap and Urbach-energy variations are likewise treated as optical descriptors rather than evidence of a specific charge-transfer or defect-mediated pathway. The data therefore point to a multifactorial composition–structure–property relationship rather than a single microscopic origin for the enhanced activity.

The roles of H_2_O_2_ and visible irradiation were further examined through the control experiments in Section 3.3.4. The control experiments showed that H_2_O_2_-assisted dark oxidation accounts for the dominant fraction of CIP removal, while visible irradiation provides a further enhancement. The observed removal is therefore more appropriately described as an H_2_O_2_-assisted, light-enhanced Fenton-like process rather than conventional semiconductor photocatalysis alone. The experiments establish an additional light-dependent contribution, but they do not resolve the underlying interfacial process.

Accordingly, 5-CuMgH is identified as the best-performing composition among those tested, rather than as a definitive Cu-content optimum. The materials represent nominal precursor formulations rather than independently quantified bulk Cu concentrations, and the number of compositions around the apparent maximum was limited. Additional compositions with narrower Cu-composition intervals would therefore be required to resolve the activity profile and determine whether a true composition-activity maximum exists.

#### 3.3.2. Effect of initial solution pH on CIP removal by 5-CuMgH

The effect of initial solution pH on CIP removal by 5-CuMgH was evaluated over a broad pH range of 3.0–12.0. The corresponding removal profiles are presented in Fig. 13, and the numerical data are summarized in Table S7 (SI). All other experimental conditions were kept constant to isolate the effect of initial solution pH.

**Fig. 13:**
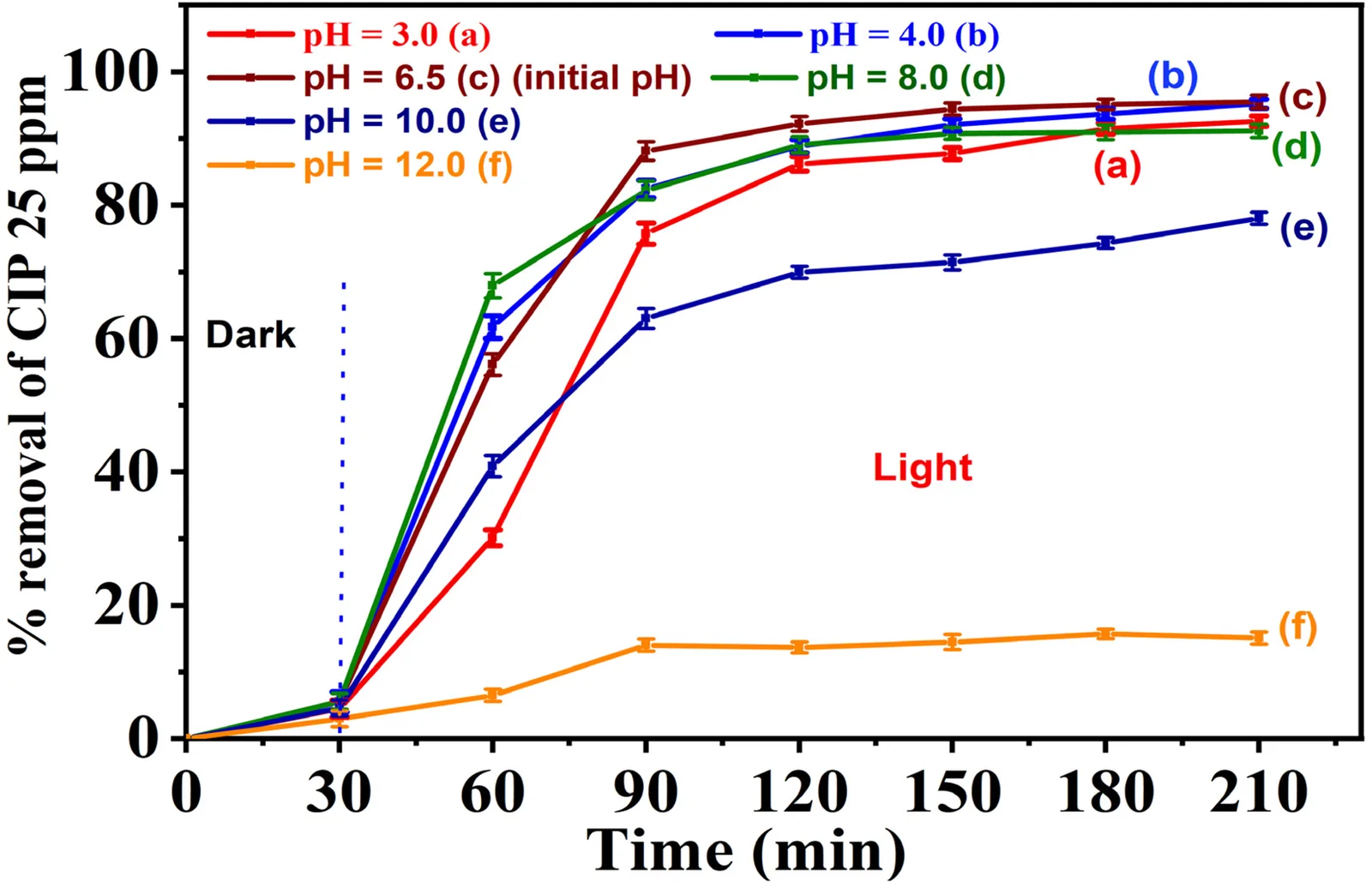
CIP (25 mg L^−1^) removal profiles over 5-CuMgH at different initial solution pH values under H_2_O_2_-assisted visible-light irradiation. The dashed line indicates the transition from the dark pre-contact stage to visible-light irradiation.

A broad favorable pH range was observed between pH 4.0 and 8.0. After 210 min of irradiation, CIP removal efficiencies of 95.2%, 95.5%, and 91.1% were obtained at pH 4.0, 6.5, and 8.0, respectively. The relatively small variation in final removal across this range indicates that high removal performance was maintained despite moderate changes in initial pH. Although the highest removal was obtained at pH 6.5, this difference should not be interpreted as evidence of a narrowly defined optimum. Rather, the results indicate that high CIP removal can be maintained over a relatively broad initial-pH range.

Statistical analysis of the final removal efficiencies at 210 min revealed a significant effect of initial solution pH (one-way ANOVA, *F*(5,12) = 9997.59, *p* = 2.80 × 10^−21^). Tukey's HSD test showed that the removal efficiencies at pH 4.0 and 6.5 were not significantly different from each other, whereas all other pairwise comparisons were statistically significant (*p* < 0.05). The corresponding Tukey groupings are provided in Table S7 (SI). These results support the high removal efficiencies observed under mildly acidic to near-neutral conditions and the substantially lower efficiencies measured under strongly alkaline initial-pH conditions.

At pH 3.0, the removal profile differed mainly during the early irradiation period. After 60 min, CIP removal reached 30.1%, compared with 61.7% and 56.1% at pH 4.0 and 6.5, respectively. This difference progressively decreased with increasing irradiation time, and the final removal reached 92.6% after 210 min. Thus, strongly acidic conditions appear to affect the initial removal kinetics more strongly than the final removal extent after prolonged treatment. The present data do not resolve the interfacial processes responsible for this slower initial response; in particular, the fresh-catalyst XPS data cannot establish changes in Cu surface chemistry during reaction.

In contrast, strongly alkaline conditions substantially inhibited CIP removal. At pH 10.0, the removal efficiency decreased to 78.1% after 210 min, whereas only 15.1% removal was achieved at pH 12.0. The strong inhibition at high pH likely reflects changes in CIP speciation and interfacial reaction conditions. Ciprofloxacin is an amphoteric molecule whose predominant aqueous speciation changes with pH, shifting from more protonated forms under acidic conditions toward zwitterionic and increasingly anionic species at higher pH. The pH-dependent speciation derived from the reported p*K*_a_ values (Fig. S6, SI) provides useful chemical context for the pronounced decrease in CIP removal under alkaline conditions. Above p*K*_a_2__ (8.89 ± 0.11), the increasing contribution of anionic CIP species may alter interfacial interactions and accessibility to reactive surface sites.

The strongly alkaline environment may also affect surface-site availability and H_2_O_2_-derived oxidation chemistry, which could further suppress removal. Similar inhibitory effects under strongly alkaline conditions have been reported for Cu-based photocatalytic systems used for antibiotic degradation, where changes in pollutant speciation and reactive oxygen species generation have been proposed as contributing factors.^32,33^ However, the present experiments do not allow the individual contributions of CIP speciation, surface interactions, and reactive-species chemistry to be separated.

Overall, the results define pH 4.0–8.0 as a broad favorable operating window, within which CIP removal remained above 90% after 210 min of irradiation. By contrast, strongly alkaline conditions, particularly pH 12.0, were clearly unfavorable. The broad pH window suggests that extensive pH adjustment may not be required when the initial solution pH falls within this range. Nevertheless, the present experiments were conducted under controlled laboratory conditions, and the influence of real-water matrix components and pH evolution during treatment requires further investigation.

#### 3.3.3. Effect of initial ciprofloxacin concentration on H_2_O_2_-assisted, light-enhanced CIP removal

The effect of initial CIP concentration on the removal performance of 5-CuMgH was evaluated over 10–100 mg L^−1^ under otherwise identical experimental conditions. The corresponding removal profiles are presented in Fig. 14, and the numerical data are summarized in Table S8 (SI).

**Fig. 14:**
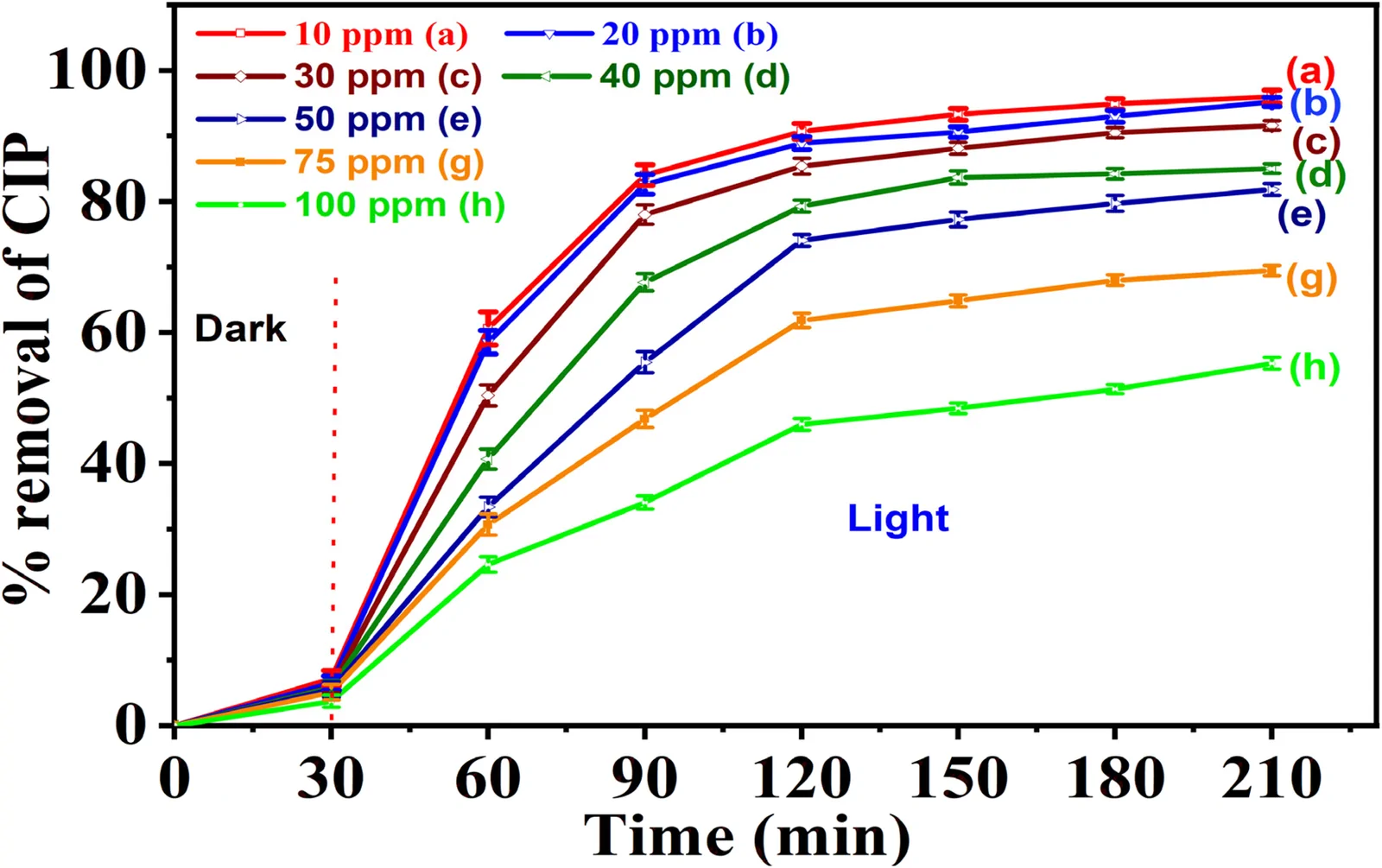
Photocatalytic degradation profiles of ciprofloxacin (CIP) over 5-CuMgH at different initial CIP concentrations under visible-light irradiation. Initial CIP concentrations: 10 mg L^−1^ (a), 20 mg L^−1^ (b), 30 mg L^−1^ (c), 40 mg L^−1^ (d), 50 mg L^−1^ (e), 75 mg L^−1^ (g), and 100 mg L^−1^ (h). The dashed line marks the transition from dark adsorption equilibrium to the irradiation stage. Error bars denote standard deviations from triplicate measurements.

A clear concentration dependence was observed. At 10 mg L^−1^, CIP removal reached 96.0% after 210 min, whereas the corresponding values at 20, 30, 40, 50, 75, and 100 mg L^−1^ were 95.2%, 91.6%, 85.0%, 81.8%, 69.5%, and 55.3%, respectively. The separation between the profiles became increasingly pronounced at concentrations above 50 mg L^−1^. Thus, increasing the initial CIP concentration progressively reduced the percentage removal achieved within the fixed treatment period.

This decrease in percentage removal should be considered together with the pollutant loading. At 10 mg L^−1^, 96.0% removal corresponds to approximately 9.6 mg L^−1^ of CIP removed, whereas 55.3% removal at 100 mg L^−1^ corresponds to approximately 55.3 mg L^−1^ removed. The lower percentage removal at higher initial concentration is therefore consistent with the greater pollutant loading imposed on a system operated at fixed catalyst loading, oxidant dose, and treatment time.

Given the low dark adsorption observed for the Cu-containing materials under the standardized pre-contact condition (Section 3.2), the concentration-dependent profiles are interpreted primarily in terms of the subsequent H_2_O_2_-assisted, light-enhanced removal process. As CIP loading increases, competition for accessible reactive sites and oxidizing species is expected to become more pronounced. Because the H_2_O_2_ dose was kept constant, the effective oxidant-to-CIP ratio also decreased progressively with increasing initial concentration. Other effects, including accumulation of reaction intermediates and attenuation of incident light, may also contribute at higher pollutant loading, but their individual contributions cannot be resolved from the present data.

The UV-vis spectra in Fig. S7 (SI) provide complementary evidence for this concentration dependence. At 10 mg L^−1^, the characteristic CIP absorption band at 271–272 nm decreased rapidly and became essentially undetectable after 90 min. At 50 mg L^−1^, the band remained detectable throughout irradiation, whereas substantial absorbance at 272 nm persisted after 210 min at 100 mg L^−1^. The persistence of this characteristic band at higher initial concentrations is consistent with the slower disappearance of CIP observed in the corresponding removal profiles.

For context, comparable removal performance has been reported for several Cu-containing visible-light-responsive systems, including Zn-doped Cu_2_O^32^ and MgIn_2_S_4_/BiOBr-based materials^34^ reported for CIP degradation. Cu(ii)-modified Mg–Al hydrotalcite/bentonite composites have likewise been investigated for visible-light-driven degradation of organic contaminants.^33^ However, direct performance comparison remains difficult because catalyst dosage, oxidant concentration, irradiation conditions, reactor configuration, and initial CIP concentration differ substantially among studies.

Overall, 5-CuMgH maintained substantial CIP removal across the investigated concentration range, although removal efficiency declined progressively with increasing initial CIP concentration. The results identify pollutant loading as an important operational variable for the present photo-assisted Fenton-like process. At higher CIP concentrations, longer irradiation and/or optimization of the H_2_O_2_ dose may therefore be needed to achieve higher removal within a fixed treatment period.

#### 3.3.4. Contributions of visible-light irradiation and H_2_O_2_ to CIP removal over 5-CuMgH

The respective contributions of visible-light irradiation and H_2_O_2_ to CIP removal over 5-CuMgH were examined using four experimental configurations: (i) 5-CuMgH under visible-light irradiation without H_2_O_2_, (ii) 5-CuMgH under visible-light irradiation in the presence of H_2_O_2_, (iii) 5-CuMgH and H_2_O_2_ under dark conditions, and (iv) H_2_O_2_ under visible-light irradiation without 5-CuMgH. The corresponding removal profiles and kinetic analyses are presented in Fig. 15, and the numerical data and statistical results are summarized in Tables S9 and S10 (SI).

**Fig. 15:**
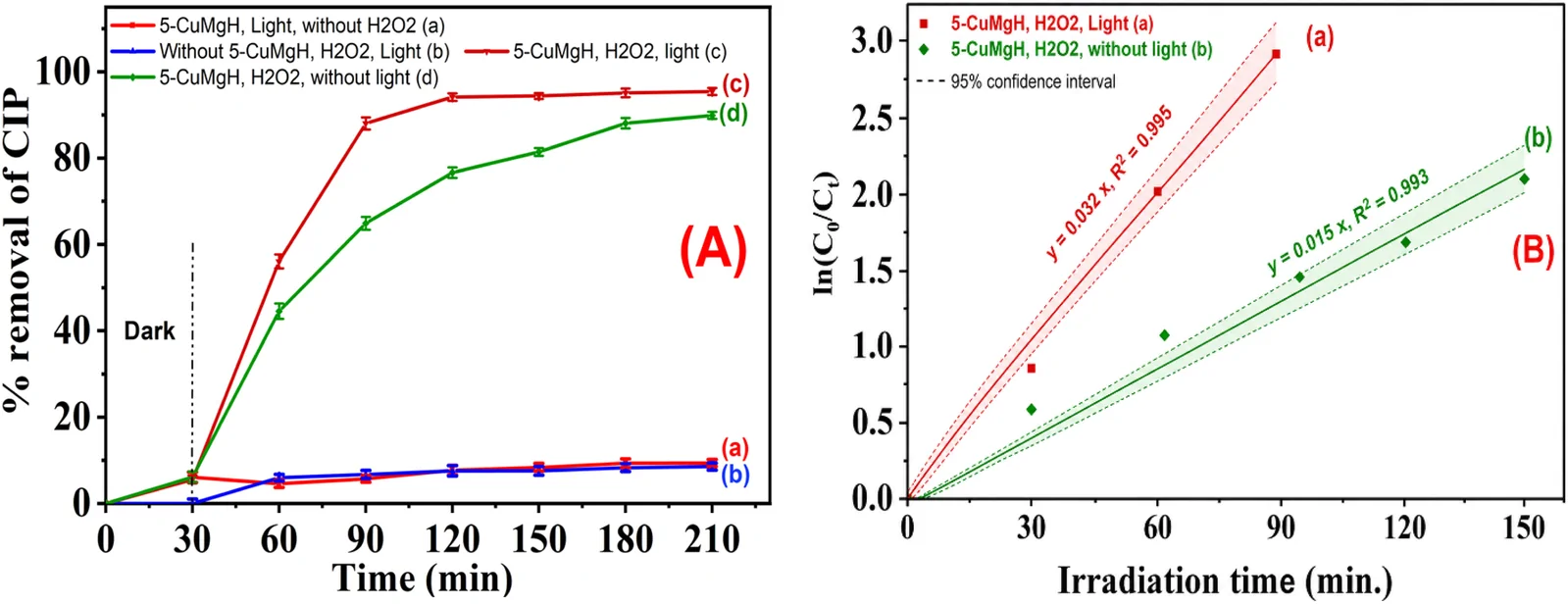
CIP removal profiles obtained under four experimental configurations: 5-CuMgH/visible light, 5-CuMgH/H_2_O_2_/visible light, 5-CuMgH/H_2_O_2_/dark, and H_2_O_2_/visible light (A), and pseudo-first-order kinetic plots for CIP removal over 5-CuMgH/H_2_O_2_ under visible-light irradiation and dark conditions (B).

The control experiments distinguished the contributions of the catalyst, H_2_O_2_, and visible irradiation. In the absence of H_2_O_2_, the 5-CuMgH/light system achieved less than 10% CIP removal throughout the reaction period, whereas H_2_O_2_ under visible irradiation without 5-CuMgH resulted in only 8.5% removal after 210 min. Thus, neither the catalyst/light-only pathway nor direct H_2_O_2_ photolysis made a substantial contribution under the investigated conditions. In contrast, 5-CuMgH in the presence of H_2_O_2_ achieved 89.9% removal after 210 min even under dark conditions, increasing to 95.5% under visible irradiation. H_2_O_2_-assisted dark oxidation therefore accounted for the dominant fraction of CIP removal, while visible irradiation provided a further enhancement.

The effect of irradiation was more pronounced in the reaction kinetics than in the final removal extent. After 90 min, CIP removal reached 88.1% under visible irradiation compared with 64.9% under dark conditions. Consistently, the apparent pseudo-first-order rate constant increased from 0.015 min^−1^ in the dark to 0.032 min^−1^ under visible irradiation, corresponding to an approximately 2.1-fold increase (*p* < 0.05; Table S10, SI). In contrast, the difference in final removal was only 5.6 percentage points. Visible irradiation therefore primarily accelerated CIP removal within the investigated reaction period rather than substantially increasing the final removal achieved after prolonged treatment.

The high activity of the 5-CuMgH/H_2_O_2_ system is consistent with Fenton-like activation of H_2_O_2_ by Cu-containing catalytic sites. Cu-based materials have previously been reported to activate H_2_O_2_ for CIP degradation through Fenton-like pathways, and visible irradiation has been shown to further enhance the kinetics of Cu-mediated H_2_O_2_ oxidation.^3,30,35^ Nevertheless, the present experiments do not establish the elementary steps responsible for H_2_O_2_ activation. XPS identified Cu^2+^ as the predominant initial surface oxidation state of the fresh catalyst, but changes in Cu oxidation state during reaction were not monitored. In addition, dissolved Cu was not quantitatively determined; consequently, a possible contribution from homogeneous Cu-mediated oxidation cannot be completely excluded. The results therefore support a Fenton-like oxidation process without requiring assignment of a specific Cu^2+^/Cu^+^ redox cycle or an exclusively heterogeneous reaction pathway.

The origin of the additional light-induced enhancement also remains unresolved. Visible irradiation may influence interfacial charge-transfer or other photoinduced processes, but the available steady-state PL, UV-vis DRS, and catalytic results do not directly characterize charge-transfer dynamics during reaction. The reactive species involved are considered separately in Section 3.4. Accordingly, the contribution of visible irradiation is interpreted here as an experimentally demonstrated kinetic enhancement rather than evidence for a specific photoinduced elementary mechanism.

The UV-vis spectral evolution in Fig. S7 (SI) is consistent with this kinetic interpretation. Under visible irradiation, the characteristic CIP absorption band at approximately 272 nm decreased more rapidly and became barely detectable after prolonged treatment, whereas its decrease was slower under dark conditions. The spectral evolution is consistent with the corresponding concentration profiles and the faster disappearance of CIP under visible irradiation.

Overall, the control experiments establish that H_2_O_2_-assisted oxidation is the dominant contributor to CIP removal, whereas visible irradiation provides an additional kinetic enhancement. Under the investigated conditions, the process is therefore more appropriately described as a photo-assisted Fenton-like process rather than conventional semiconductor photocatalysis. The experiments demonstrate a measurable contribution of visible irradiation to the reaction kinetics, whereas the molecular origin of this enhancement and the possible contribution of dissolved Cu remain unresolved.

#### 3.3.5. Comparison with representative visible-light-driven photocatalytic systems

A quantitative comparison of 5-CuMgH with representative visible-light-driven systems reported for CIP degradation is presented in Table 4. The comparison includes catalyst dosage, initial CIP concentration, light source and wavelength, irradiation intensity where available, CIP removal/degradation efficiency, irradiation time, and apparent pseudo-first-order rate constant (*k*_app_). Where both catalyst dosage and *k*_app_ were reported, the corresponding mass-normalized value (*k*_app_/*C*_cat_) was calculated as an auxiliary comparative index.

**Table 4:** Comparison of representative visible-light-driven photocatalysts for CIP degradation under the reported experimental conditions^a^

Material	Light source/wavelength/intensity	Catalyst dosage (g L^−1^)	Initial CIP (mg L^−1^)	CIP removal/degradation (%)	Irradiation time (min)	*k*_app_ (min^−^)	Mass-normalized *k*_app_ (L g^−1^ min^−1^)	Ref.
FeWO_4_/NC-800	Xe lamp, *λ* > 420 nm	0.08	20	92.23	100	0.0240	0.300	36
2% C/BiOBr	500 W Xe lamp	1.00	10	96.8	60	0.0435	0.0435	37
Cu_*x*_O/MOF	Visible light, 465 ± 40 nm	0.50	15	≈92	120	0.0480	0.0960	38
g-C_3_N_4_/BiOCl-2 + PS	300 W Xe lamp, simulated visible light	0.30	10	NR	90	0.0215	0.0717	39
BiOBr/30%MgIn_2_S_4_	100 W Xe solar lamp, 10 cm	0.80	10	89.9	60	0.0310	0.0388	34
**5-CuMgH**	**30 W Philips LED, *λ***_**max**_ ≈ **464 nm**	**0.80**	**25**	**95.5**	**210**	**0.0320**	**0.0400**	**This work**

a *k*_app_, apparent pseudo-first-order rate constant; mass-normalized *k*_app_ = *k*_app_/*C*_cat_, where *C*_cat_ is total catalyst loading. NR, not reported. Normalized values are comparative indices, not intrinsic catalytic activities.

Under the respective experimental conditions, the 5-CuMgH/H_2_O_2_/visible-light system achieved 95.5% CIP removal after 210 min, with an apparent *k*_app_ of 0.0320 min^−1^ under irradiation from a 30 W LED. The obtained *k*_app_ is of the same order as those reported for representative visible-light systems, including FeWO_4_/NC-800 (0.0240 min^−1^),^36^ g-C_3_N_4_/BiOCl-2 + PS (0.0215 min^−1^),^39^ and BiOBr/30%MgIn_2_S_4_ (0.0310 min^−1^),^34^ while remaining below the values reported for 2% C/BiOBr (0.0435 min^−1^)^37^ and Cu_*x*_O/MOF (0.0480 min^−1^).^38^ Thus, the kinetic performance of 5-CuMgH falls within the range reported for the selected visible-light-responsive systems rather than indicating a universally superior activity.

The 95.5% CIP removal achieved by 5-CuMgH is likewise comparable in magnitude to the reported values for several representative systems. However, this comparison must be considered in the context of differences in catalyst loading, initial CIP concentration, irradiation time, and irradiation conditions. For example, 2% C/BiOBr achieved 96.8% CIP degradation within 60 min at an initial CIP concentration of 10 mg L^−1^, whereas BiOBr/30%MgIn_2_S_4_ achieved 89.9% degradation within the same irradiation period under its reported experimental conditions.^1–5^ These differences illustrate why removal efficiency alone cannot be used to establish a direct performance ranking across the literature systems.

Where both *k*_app_ and catalyst loading were available, *k*_app_/*C*_cat_ was additionally calculated as an auxiliary index of the apparent kinetic response relative to catalyst loading. For 5-CuMgH, the normalized value was 0.0400 L g^−1^ min^−1^, which is comparable to that of BiOBr/30%MgIn_2_S_4_ (0.0388 L g^−1^ min^−1^), but lower than those reported for FeWO_4_/NC-800 (0.300 L g^−1^ min^−1^), Cu_*x*_O/MOF (0.0960 L g^−1^ min^−1^), and g-C_3_N_4_/BiOCl-2 + PS (0.0717 L g^−1^ min^−1^).^1–5^ Where either catalyst loading or *k*_app_ was unavailable, the normalized value was not calculated and should be reported as NR. These normalized values remain condition-dependent comparative indices, because catalyst-mass normalization does not account for differences in photon flux, irradiation wavelength, reactor geometry, catalyst optical properties, adsorption contributions, or oxidant chemistry.

The irradiation conditions also warrant careful consideration. The present study employed a 30 W nominal electrical-power LED with *λ*_max_ ≈ 464 nm, whereas the literature systems used different light sources and configurations, including Xe lamps and visible-light irradiation. The nominal electrical power of the present LED should therefore not be interpreted as an irradiance value. Because irradiance was not directly measured, it is reported as NR (not measured) rather than being inferred from the nominal lamp power. Differences in lamp-to-reactor distance, illuminated area, reactor geometry, spectral output, and photon flux may substantially influence the observed reaction kinetics even when nominal lamp powers appear similar.

The chemical basis of the compared systems also differs. Several literature systems rely primarily on semiconductor photocatalysis, whereas the present system operates through H_2_O_2_-assisted, light-enhanced Fenton-like oxidation. As demonstrated by the control experiments in Section 3.3.4, CIP removal by 5-CuMgH/H_2_O_2_ reached 89.9% under dark conditions and increased to 95.5% under visible-light irradiation, whereas the catalyst/light-only system and H_2_O_2_/light system produced less than 10% removal and 8.5% removal, respectively. Thus, H_2_O_2_-assisted oxidation contributes substantially to the overall removal, while visible irradiation provides an additional kinetic enhancement. Accordingly, the *k*_app_ values listed in Table 4 should be interpreted as condition-dependent kinetic parameters rather than directly comparable measures of intrinsic photocatalytic activity across different reaction systems.

In conclusion, the comparison supports the competitive performance of 5-CuMgH under the specific experimental conditions investigated, but does not establish universal superiority over the selected literature materials. A rigorous comparison of catalytic efficiency would require harmonized experiments under identical CIP concentration, catalyst loading, oxidant dose, photon flux, reactor geometry, and analytical conditions.

### 3.4. Reactive-species trapping experiments and proposed mechanism of CIP degradation over 5-CuMgH

Reactive-species trapping experiments were performed to examine the reactive species potentially involved in CIP transformation over 5-CuMgH under visible-light irradiation. Ascorbic acid (AA, 1 mM), isopropyl alcohol (IPA, 10 mM and concentrated IPA), and EDTA-2Na (1 mM) were used as scavengers nominally targeting ˙O_2_^−^, ˙OH, and photogenerated holes (h^+^), respectively. The corresponding removal profiles are shown in Fig. 16, and the quantitative results are summarized in Table S11 (SI).

**Fig. 16:**
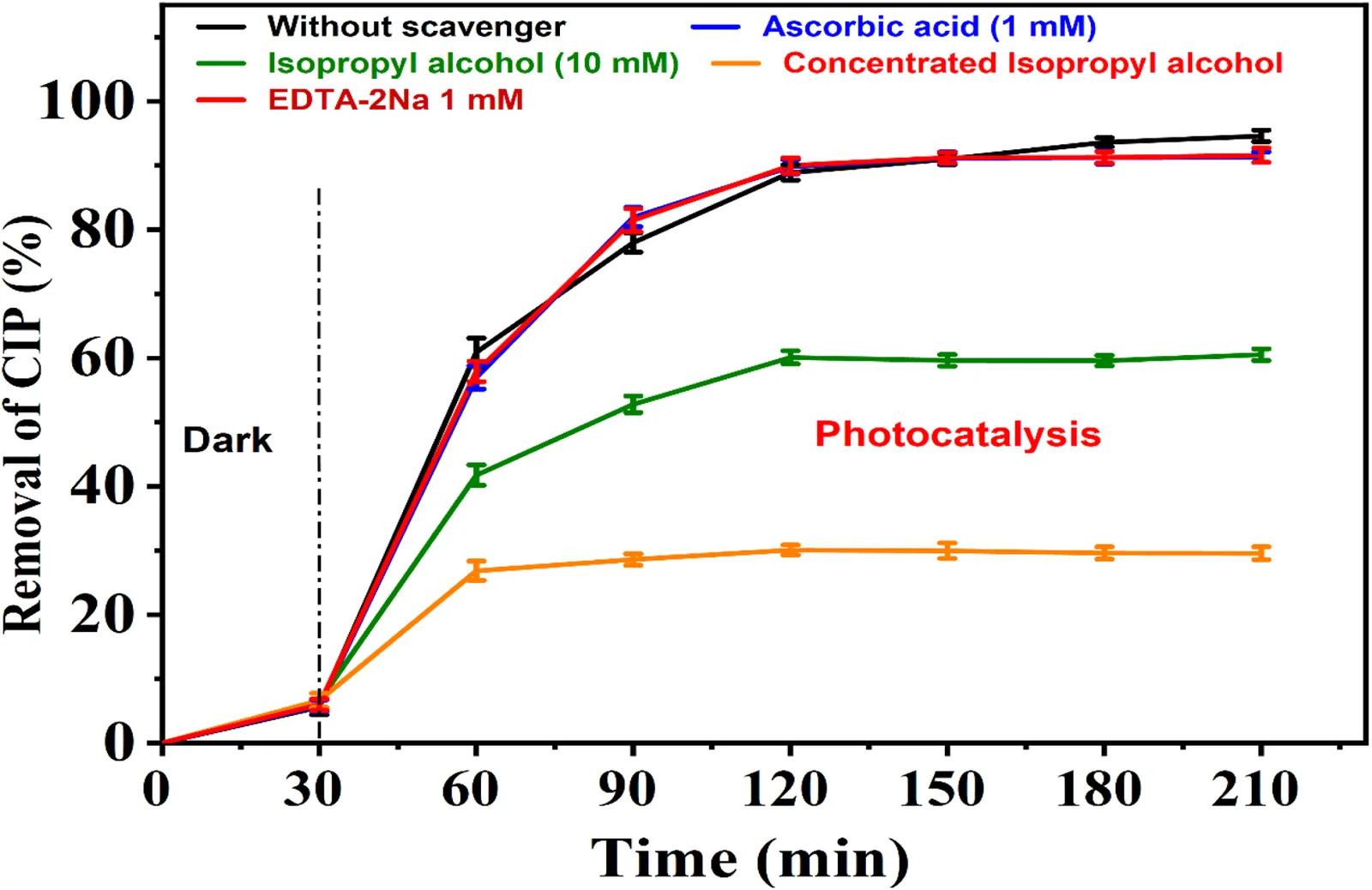
Reactive-species trapping experiments for CIP transformation over 5-CuMgH under visible-light irradiation. Ascorbic acid (AA, 1 mM), isopropyl alcohol (IPA, 10 mM and concentrated IPA), and EDTA-2Na (1 mM) were used as scavengers nominally targeting ˙O_2_^−^, ˙OH, and h^+^, respectively. Error bars represent standard deviations (*n* = 3).

In the absence of a scavenger, CIP removal reached 94.6% after 210 min. Addition of AA reduced the removal only slightly, to 91.3 ± 2.4%, while EDTA-2Na resulted in 85.9 ± 2.7% removal. In contrast, IPA markedly suppressed CIP removal, decreasing the removal to 60.5 ± 2.8% at 10 mM and 29.6 ± 2.6% with concentrated IPA. The pronounced and concentration-dependent inhibition by IPA is consistent with an important apparent contribution of ˙OH to CIP transformation under the present conditions.

Because scavenger selectivity is not absolute in heterogeneous oxidation systems, these experiments provide indirect evidence for reactive-species involvement rather than definitive identification or quantitative determination of individual ROS. The relatively small inhibition observed with AA and EDTA-2Na is consistent with less pronounced apparent contributions from ˙O_2_^−^ and h^+^, respectively, whereas the stronger inhibition produced by IPA supports a more substantial role of ˙OH. Singlet oxygen (^1^O_2_) was not examined in the present study. Direct EPR spin-trapping measurements would therefore be required to establish the identity and relative contributions of individual reactive species.

The control experiments in Section 3.3.4 help distinguish the roles of H_2_O_2_ and visible irradiation. The 5-CuMgH/light system produced less than 10% CIP removal in the absence of H_2_O_2_, while H_2_O_2_ under visible irradiation without 5-CuMgH resulted in only 8.5% removal after 210 min. By comparison, the 5-CuMgH/H_2_O_2_ system achieved 89.9% removal under dark conditions and 95.5% under visible irradiation. H_2_O_2_-assisted oxidation therefore accounts for the major contribution to CIP removal, whereas visible irradiation provides an additional kinetic enhancement. The substantial dark activity is consistent with H_2_O_2_ activation associated with the Cu-containing material and a Fenton-like oxidation process; however, the elementary steps responsible for H_2_O_2_ activation cannot be resolved from the present measurements.

The high activity of 5-CuMgH occurs together with changes in its crystallographic, textural, optical, and surface-chemical characteristics. In particular, 5-CuMgH exhibits an apparent band gap of 1.86 eV and an Urbach energy of 3.41 eV, together with substantially modified textural properties relative to the lower-Cu compositions. The activity trend therefore cannot be attributed to a single material descriptor. Notably, although 6-CuMgH possesses a higher BET surface area, its CIP-removal performance is lower than that of 5-CuMgH, indicating that surface area alone is insufficient to explain the composition–activity relationship. Recent XPS-based studies have likewise shown that complementary surface spectroscopic analyses can reveal dopant-induced changes in the local chemical and electronic environment, providing additional insight into composition-dependent catalytic behavior.^40^

The proposed transformation scheme is summarized in Fig. 17. XPS identified Cu^2+^ as the predominant surface oxidation state of fresh 5-CuMgH. Cu-containing surface sites may therefore participate in H_2_O_2_ activation and subsequent oxidation. However, because the Cu oxidation state was not monitored after reaction, the present data do not establish Cu redox evolution during catalysis; accordingly, no specific Cu^2+^/Cu^+^ redox cycle is proposed. Dissolved Cu was also not quantitatively determined, and a possible contribution from homogeneous Cu-mediated oxidation cannot therefore be completely excluded. The process is consequently described as a Cu-containing-material-assisted Fenton-like oxidation, without assuming that H_2_O_2_ activation occurs exclusively at intact heterogeneous surface sites.

**Fig. 17:**
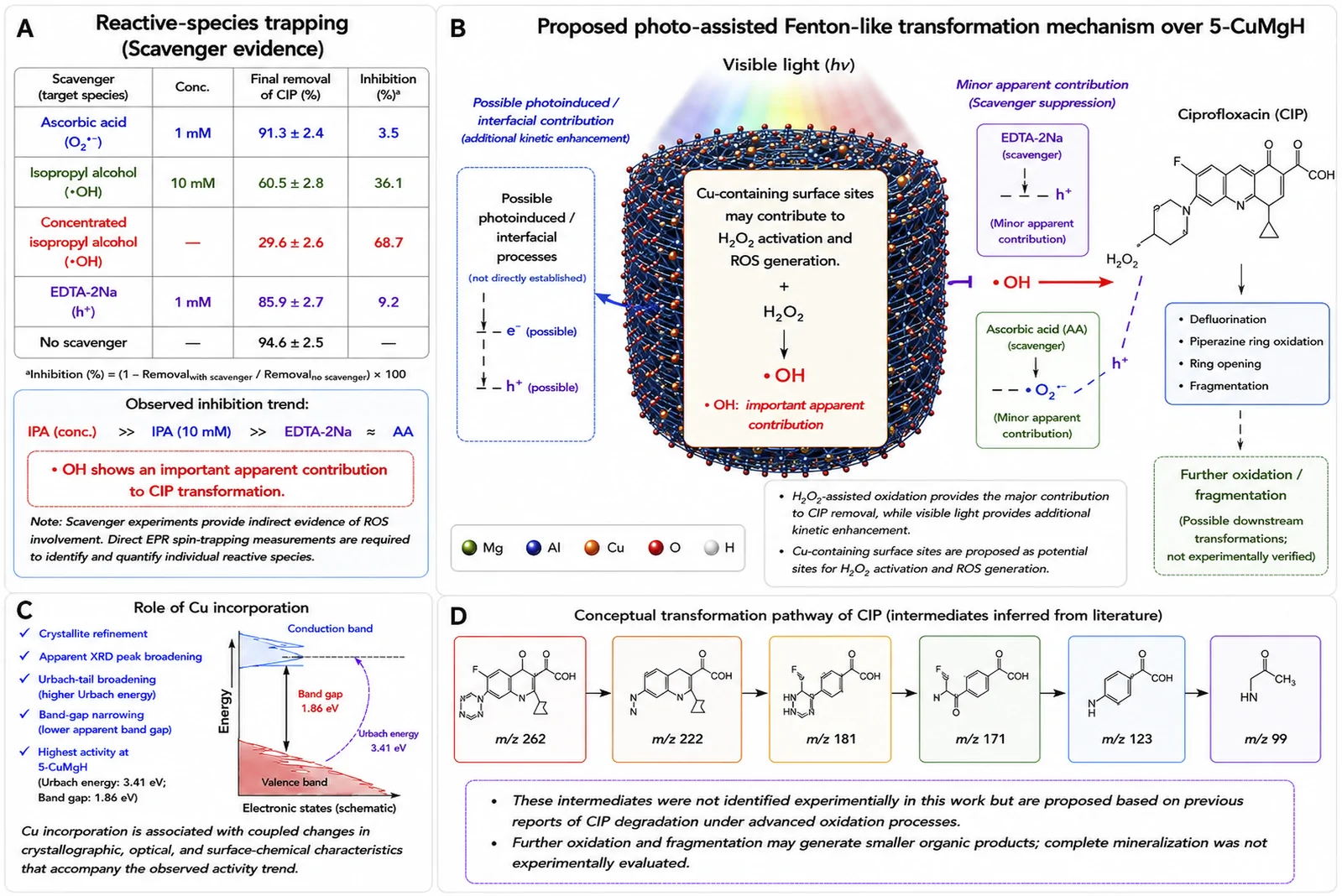
Proposed photo-assisted Fenton-like mechanism and CIP transformation over 5-CuMgH under H_2_O_2_-assisted visible-light irradiation: (A) scavenger evidence for reactive-species contributions; (B) proposed H_2_O_2_ activation and ROS-generation mechanism; (C) conceptual role of Cu incorporation in the structural and optical properties of 5-CuMgH; and (D) proposed CIP transformation pathway based on previously reported intermediates.

Although XRD peak broadening and increased Urbach energy accompany Cu incorporation, these parameters provide indirect information on crystallographic coherence and electronic band-tail states, respectively, rather than direct evidence of local lattice distortion or short-range structural heterogeneity. Post-reaction XPS, Cu LMM Auger spectroscopy or XANES, together with local-structure techniques such as HRTEM or total-scattering pair-distribution-function analysis, would provide more direct evidence regarding Cu-state evolution and local structural characteristics. Such analyses were beyond the scope of the present study.

Visible irradiation further increased the apparent reaction rate, with the pseudo-first-order rate constant increasing from 0.015 min^−1^ under dark H_2_O_2_-assisted conditions to 0.032 min^−1^ under visible irradiation (Section 3.3.4). The steady-state PL and UV-vis DRS results characterize the optical and emissive properties of 5-CuMgH, but do not directly demonstrate enhanced charge separation, carrier lifetime, or a defined interfacial charge-transfer pathway. The light-induced contribution is therefore interpreted as an experimentally demonstrated kinetic enhancement, without assigning a specific photoinduced elementary mechanism.

If ˙OH contributes substantially to CIP transformation, it may attack chemically susceptible sites within the piperazine and quinolone moieties, potentially promoting defluorination, heterocyclic cleavage, ring opening, and molecular fragmentation. Previous studies of CIP degradation under advanced oxidation conditions have reported transformation products with representative *m*/*z* values of 262, 222, 181, 171, 123, and 99. These species are included in Fig. 17 as literature-supported possible transformation products, rather than as intermediates experimentally identified in the present system.

Further oxidation and fragmentation of such products may generate smaller organic compounds. However, the present study evaluated CIP removal rather than complete mineralization; transformation products, TOC/DOC removal, and inorganic carbon formation were not directly measured. The pathway shown in Fig. 17 should therefore be regarded as a plausible transformation scheme rather than a verified sequence of molecular intermediates. Time-resolved LC-MS/MS analysis would be required to establish the formation and evolution of individual transformation products.

Together, the results support a multifactorial, H_2_O_2_-assisted, visible-light-enhanced Fenton-like process in which H_2_O_2_-mediated oxidation provides the major contribution to CIP removal and visible irradiation increases the apparent reaction rate. The scavenging experiments indicate an important apparent contribution of ˙OH, while the composition-dependent structural, optical, textural, and surface-chemical changes suggest that the catalytic behavior arises from several interacting material characteristics rather than from surface area or any single descriptor. The molecular transformation scheme is constrained by reported CIP degradation chemistry and the present kinetic and scavenging results; it should therefore be regarded as an evidence-constrained mechanistic interpretation rather than a directly resolved molecular pathway.

### 3.5. Stability and reusability of 4-CuMgH during photocatalytic degradation of ciprofloxacin

Although 5-CuMgH exhibited the highest CIP removal among the investigated compositions, the recycling experiment was conducted using 4-CuMgH because insufficient 5-CuMgH remained after the preceding screening and characterization experiments. Accordingly, the following results describe the repeated-use behavior of 4-CuMgH specifically and should not be interpreted as evidence that 4-CuMgH was the optimal composition.

The catalyst maintained a high level of photocatalytic activity throughout the recycling experiments (Fig. 18A). After 180 min of visible-light irradiation, the CIP removal efficiency decreased gradually from 94.1% during the first cycle to 88.8%, 85.9%, 84.1%, and 79.5% during the second, third, fourth, and fifth cycles, respectively. Despite this gradual decline, 84.5% of the initial photocatalytic activity was retained after five consecutive reuse cycles, reflecting good operational stability under the investigated conditions. A similar tendency was observed at 120 min irradiation time (Fig. S8 (SI)), where the catalyst preserved 78.1% of its initial activity after the fifth cycle. The gradual decrease in photocatalytic performance, despite the overall preservation of the catalyst structure and textural characteristics discussed below, suggests that deactivation may involve processes that are not readily resolved by XRD or N_2_ adsorption–desorption analysis.

**Fig. 18:**
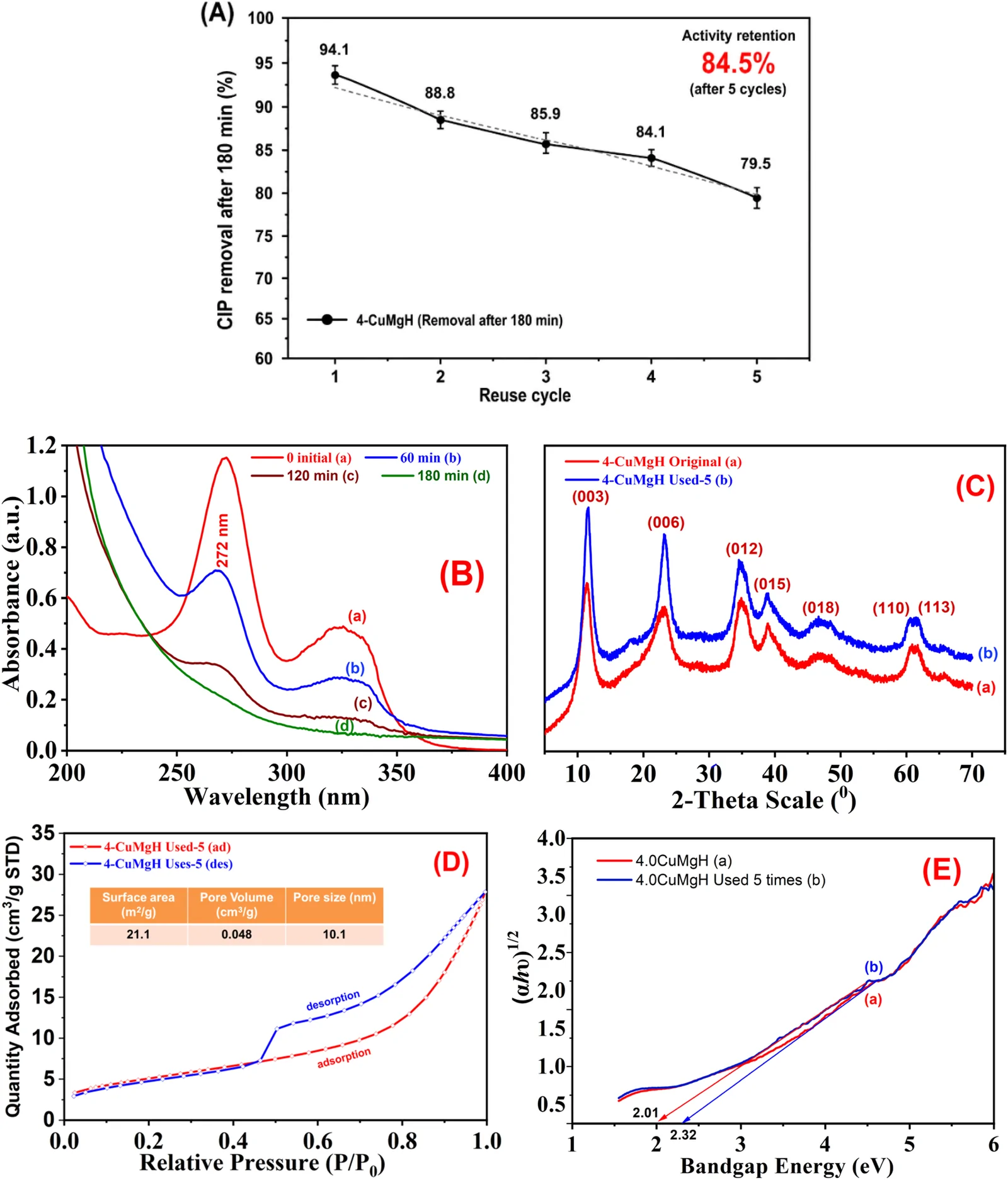
Characterization and reusability of 4-CuMgH. (A) CIP removal efficiency over five reuse cycles, showing 84.5% activity retention. (B) UV-vis absorption spectra of CIP over time. (C) XRD patterns of 4-CuMgH before and after five cycles. (D) N_2_ adsorption–desorption isotherms and pore properties of used 4-CuMgH. (E) Tauc plots determining the bandgap energy of 4-CuMgH samples.

Surface-related processes, including the accumulation of strongly adsorbed CIP molecules, transformation intermediates, or other surface-bound species, may plausibly contribute to the observed activity loss. However, this interpretation remains tentative because post-reaction XPS or FTIR characterization was not performed in the present study and, therefore, the accumulation of such surface species cannot be directly verified. Minor catalyst loss during the recovery and washing procedures may also contribute to the apparent decrease in activity. The photocatalytic effectiveness of the reused catalyst remained evident during the fifth cycle (Fig. 18B). The absorption band of ciprofloxacin at 272 nm steadily diminished with irradiation time and was almost completely suppressed after 180 min, matching the high degradation efficiency recorded in (Fig. 18A).

XRD patterns collected after five cycles (Fig. 18C) closely resembled those of the fresh material. All characteristic LDH reflections, including the (003), (006), (012), (015), (018), (110), and (113) planes, were preserved, while no new crystalline phases emerged. These features point to a stable hydrotalcite framework with no detectable phase transformation during repeated operation. Thus, within the resolution of XRD, the observed activity loss is unlikely to be primarily associated with major crystallographic degradation or phase transformation of the LDH framework.

The reused catalyst retained a type IV isotherm with an H3 hysteresis loop (Fig. 18D). A BET surface area of 21.1 m^2^ g^−1^, compared with 22.6 m^2^ g^−1^ for the fresh sample, reflects only minor textural change after five cycles. Pore volume (0.048 cm^3^ g^−1^) and average pore diameter (10.1 nm) remained within the same range, consistent with preservation of the mesoporous structure. Together with the XRD results, these data indicate that the major structural and textural characteristics of the catalyst were largely preserved after repeated operation. Nevertheless, these techniques cannot directly resolve changes in surface chemical states or the accumulation of strongly bound surface species.

Optical characteristics were also largely retained (Fig. 18E). The band gap decreased from 2.32 to 2.01 eV after repeated use, possibly owing to subtle modifications in the local Cu environment or adsorption of residual surface species. This possible contribution of residual surface species should be regarded as a plausible interpretation rather than direct experimental evidence, because post-reaction surface-sensitive characterization was not available. Despite this shift, visible-light absorption remained strong. More importantly, photocatalytic activity was sustained throughout the recycling tests, indicating that the observed optical changes did not impair catalyst functionality.

The recycling experiments and post-reaction characterization consistently depict a material that remains structurally intact, texturally stable, and photocatalytically active after repeated use. Retention of 84.5% of the initial activity after five cycles supports the suitability of 4-CuMgH for visible-light-driven treatment of ciprofloxacin-contaminated water. Nevertheless, the progressive activity loss cannot be assigned conclusively to any specific deactivation mechanism based on the present characterization. In particular, the possible contribution of surface-bound species remains experimentally unresolved and would require post-reaction XPS and/or FTIR analysis of the used catalyst for direct verification.

### 3.6. Limitations and future perspectives

The present study provides experimental evidence for composition-dependent relationships among the structural, optical, surface-chemical, and catalytic properties of Cu-substituted MgAl-LDH, although several mechanistic aspects remain unresolved. The remaining limitations are prioritised according to their relevance to the principal conclusions.

The highest priority is to clarify ROS involvement and Cu-related surface chemistry. The scavenger experiments provide qualitative rather than definitive evidence for ROS involvement, while XPS identifies Cu^2+^ only in the fresh catalyst. EPR spin trapping would provide a more direct test of ROS involvement, while post-reaction XPS/Cu LMM analysis could assess changes in the surface Cu chemical state. Quantitative dissolved-Cu measurements would additionally assess the extent of possible Cu leaching.

A second priority is to clarify the CIP transformation pathway and the extent of mineralization. CIP disappearance alone does not identify transformation products or demonstrate complete mineralization. Time-resolved LC-MS/MS, complemented by TOC/DOC analysis, would therefore provide critical evidence for characterizing transformation products and assessing the extent of mineralization.

The photophysical contribution remains a further mechanistic uncertainty. PL measurements were obtained only for MgAlH and 5-CuMgH and therefore do not establish a continuous photophysical trend across the Cu-substituted series. Time-resolved PL, transient photocurrent, and EIS could further assess carrier dynamics and the contribution of visible irradiation.

The adsorption mechanism and local structural changes are less directly resolved. Zeta-potential measurements were available only for MgAlH and 5-CuMgH, while XRD peak broadening and Urbach energy do not independently establish local lattice distortion. Complementary FTIR analysis of CIP-loaded catalysts and local-structure characterization could refine these interpretations.

Catalyst recycling also remains a practical limitation. Although XRD and BET indicate that the structure and textural properties of 4-CuMgH were largely preserved after reuse, the activity loss may be associated with residual surface-bound species. However, this remains unconfirmed because post-reaction XPS or FTIR analyses were not performed. Future recycling studies could evaluate ethanol- or methanol-washing followed by activity reassessment to examine whether partial activity recovery is consistent with a contribution from surface-bound species.

Finally, the experiments were conducted in a model CIP solution under controlled laboratory conditions. Natural organic matter, competing ions, pH variation, and incident irradiance may affect H_2_O_2_ consumption, ROS availability, and the light-assisted contribution. Studies in realistic water matrices and under calibrated irradiance would therefore be needed to assess practical transferability.

Overall, the present evidence supports H_2_O_2_-assisted Fenton-like oxidation and shows an additional increase in the apparent reaction rate under visible irradiation, while the scavenger results provide qualitative evidence for a contribution from ˙OH. Direct evidence on ROS involvement, post-reaction Cu/surface chemistry, and transformation products therefore remains the highest priority for future mechanistic studies.

## 4. Conclusions

Cu substitution provides a composition-dependent means of modifying the structural, optical, surface-chemical, and textural characteristics of MgAl-LDHs, accompanied by changes in their oxidative reactivity toward ciprofloxacin. Within the investigated composition range, 5-CuMgH showed the highest activity, coinciding with the lowest apparent band gap (1.86 eV) and highest Urbach energy (3.407 eV). These correlations support an association between Cu-induced changes in material properties and catalytic behavior, but do not establish a causal role for band-gap narrowing or disorder-related features alone.

Under H_2_O_2_-assisted visible-light irradiation, 5-CuMgH achieved 95.5% CIP removal after 210 min, with an apparent pseudo-first-order rate constant of 0.032 min^−1^. Its high activity under dark H_2_O_2_ conditions (89.9%) indicates that H_2_O_2_-assisted Fenton-like oxidation is the major contributor to CIP removal, while visible irradiation provides an additional kinetic enhancement. The scavenging results further implicate ˙OH, although the limited selectivity of the probes prevents definitive assignment of individual ROS contributions. The lower activity of 6-CuMgH indicates an apparent activity maximum within the investigated composition range, but not a definitive Cu-content optimum.

The results therefore indicate that catalytic behavior is associated with the concurrent evolution of crystallographic, optical, surface-chemical, and textural characteristics rather than with any single descriptor. Optical changes thus appear to accompany, rather than independently account for, the observed H_2_O_2_-assisted and light-enhanced reactivity of the Cu-substituted LDHs. Resolving the underlying Cu-state evolution, reactive-species chemistry, electronic dynamics, and molecular transformation pathways will require complementary *operando*, spectroscopic, computational, and time-resolved measurements, together with validation in realistic water matrices.

## Author contributions

V. N. V.; methodology: V. N. V., N. T. T. L. and T. H. T. N.; software: T. T. H. N., T. T. L. N. and T. M. V. N.; validation: V. N. V., T. T. L. N. and T. T. H. N.; data curation: V. N. V., T. T. H. N. and T. M. V. N.; writing original draft preparation: T. X. V. and V. N. V.; writing – review and editing, V. T. X.; visualization: V. N. V., T. X. V., and T. M. V. N.

## Conflicts of interest

There are no conflicts to declare.

## Supplementary Material

RA-016-D6RA05478A-s001

## Data Availability

All data supporting the findings of this study are available within the article and its supplementary information (SI). The datasets generated and analysed during the current study, including material synthesis parameters, photocatalytic performance data, adsorption experiments, optical characterization data, and catalyst reusability results, are provided in the manuscript and SI. Additional data supporting the findings of this work are available from the corresponding author upon reasonable request (xuanvt@tnus.edu.vn). Supplementary information: Table S1, summarizing precursor compositions and reagent amounts used for synthesis; Table S2, providing CIP calibration concentrations and corresponding absorbance values; Table S3, reporting statistical analyses of the optical band-gap and Urbach energies; Tables S4–S5, presenting time-dependent dark adsorption and visible-light photocatalytic removal data; Tables S6–S7, summarizing kinetic and statistical analyses and the effect of initial pH; and Tables S8–S11, covering the effects of initial CIP concentration, control experiments, reaction kinetics under different conditions, and reactive-species scavenging tests. Fig. S1–S3 show the CIP calibration curve, Tauc plots, and Urbach plots, while Fig. S4–S8 present zeta potentials, calculated CIP speciation, UV-vis spectral evolution, and catalyst reusability data. See DOI: https://doi.org/10.1039/d6ra05478a.
