# Supplementary material for: Cu2+ Substitution modulates the structural and optical properties of MgAl hydrotalcites for photo-assisted Fenton-like degradation of ciprofloxacin
Source: RSC Adv. 2026 Sep 14;16(44):55026–51. doi: 10.1039/d6ra05478a (PMC13573863; doi:10.1039/d6ra05478a)
Supplement: Supplementary file 1 [file RA-016-D6RA05478A-s001.pdf]

This journal is © The Royal Society of Chemistry 2026

SUPPLEMENTARY INFORMATION

Title: Cu^{2+} Substitution Modulates the Structural and Optical Properties of MgAl Hydrotalcites for Photo-Assisted Fenton-like Degradation of Ciprofloxacin

Table S1. Precursor compositions and reagent masses used for the synthesis of MgAlH and Cu-substituted hydrotalcite materials (n-CuMgH).

Sample	Cu:Mg:Al:CO_3^{2-} molar ratio	$Cu(NO_3)_2·3H_2O$ (g)	$Mg(NO_3)_2·6H_2O$ (g)	$Al(NO_3)_3·9H_2O$ (g)	Na_2CO_3 (g)
MgAlH	0:7.0:3.0:1.5	0.00	17.92	11.25	1.59
2-CuMgH	2.0:5.0:3.0:1.5	4.84	12.80	11.25	1.59
3-CuMgH	3.0:4.0:3.0:1.5	7.26	10.24	11.25	1.59
4-CuMgH	4.0:3.0:3.0:1.5	9.68	7.68	11.25	1.59
5-CuMgH	5.0:2.0:3.0:1.5	12.10	5.12	11.25	1.59
6-CuMgH	6.0:1.0:3.0:1.5	14.52	2.56	11.25	1.59

Table 2. Calibration concentrations of ciprofloxacin (CIP) and corresponding absorbance values measured at 272 nm.

CIP concentration (mg L^{-1})	1.0	2.0	4.0	6.0	8.0	10.0	12.0	15.0
Absorbance (a.u.)	0.077	0.180	0.369	0.550	0.731	0.910	1.089	1.336

Table S3. Descriptive statistics, 95% confidence intervals, one-way ANOVA, and Tukey HSD grouping for *E*g and *E*u.

Sample	***E*g (eV), mean ± SD**	**95% CI**	**Tukey group**	***E*u (eV), mean ± SD**	**95% CI**	**Tukey group**
MgAlH	3.550 ± 0.040	3.451–3.649	**a**	0.347 ± 0.010	0.322–0.372	**f**
2-CuMgH	2.680 ± 0.035	2.593–2.767	**b**	0.983 ± 0.025	0.921–1.045	**e**
3-CuMgH	2.520 ± 0.035	2.433–2.607	**c**	1.758 ± 0.040	1.659–1.857	**d**
4-CuMgH	2.060 ± 0.035	1.973–2.147	**e**	3.142 ± 0.060	2.993–3.291	**b**
5-CuMgH	1.860 ± 0.035	1.773–1.947	**f**	3.407 ± 0.065	3.246–3.569	**a**
6-CuMgH	2.420 ± 0.035	2.333–2.507	**d**	1.416 ± 0.035	1.329–1.503	**c**
**ANOVA, $F(5,12)$**	**812.53**	—	—	**2310.54**	—	—
***p*-value**	**9.53×10^{-15}**	—	—	**1.83×10^{-17}**	—	—

Values are presented as mean ± SD (n = 3). The 95% confidence intervals (CIs) were calculated from the replicate-level parameter estimates. Different lowercase letters indicate statistically significant differences among samples according to one-way ANOVA followed by Tukey's HSD test at α = 0.05.

Table S4. Time-dependent adsorption efficiencies (%) of ciprofloxacin (25 mg L^{-1}) under dark conditions for MgAlH and Cu-substituted hydrotalcite materials (n-CuMgH).

Contact time (min)	Average adsorption efficiency of CIP (25 mg L^{-1}) (%)					
	MgAlH	2-CuMgH	3-CuMgH	4-CuMgH	5-CuMgH	6-CuMgH
0	0.0	0.0	0.0	0.0	0.0	0.0
30	59.4 ± 0.78[a]	8.4 ± 0.46[b]	6.3 ± 0.36[c]	5.9 ± 0.36[c]	5.8 ± 0.20[c]	5.5 ± 0.36[c]
60	62.2	9.3	7.0	6.9	6.1	4.0
90	63.0	8.4	6.2	5.5	5.8	3.4
120	62.3	7.8	5.2	4.3	4.8	2.7
150	60.9	6.3	4.9	3.1	3.6	2.8
180	59.8	6.1	4.8	2.9	4.0	3.0

Values are presented as mean ± SD (n = 3). For statistical comparison, adsorption efficiencies at the standardized 30 min dark pre-contact condition were analyzed by one-way ANOVA followed by Tukey's HSD test at $\alpha = 0.05$. Different superscript lowercase letters within the 30 min row indicate statistically significant differences among the materials. One-way ANOVA: $F(5,12) = 6762.32$, $p = 2.92 \times 10^{-20}$.

Table S5. Average ciprofloxacin (CIP, 25 mg L^{-1}) removal efficiencies of the synthesized materials under LED-light irradiation

Time (min)	Average CIP removal efficiency (%)					
	**MgAlH**	**2-CuMgH**	**3-CuMgH**	**4-CuMgH**	**5-CuMgH**	**6-CuMgH**
0	0	0	0	0	0	0
30_{ad}	61.1	8.2	5.9	6.4	5.6	4.4
60	21.5	19.8	28.7	42.2	56.1	44.6
90	23.8	50.7	63.0	80.7	88.1	64.1
120	20.6	72.8	81.0	89.3	92.2	85.9
150	23.7	82.5	88.5	93.8	94.4	92.4
180	24.5	90.4	92.4	94.6	95.1	93.9
210	24.7	94.0	95.3	95.5	95.5	94.0

Note: ^{ad} Adsorption efficiency measured after 30 min of dark equilibration prior to light irradiation.

Table S6. Regression-derived pseudo-first-order rate constants (k), 95% confidence intervals, goodness-of-fit (R^2), and Tukey HSD grouping for CIP degradation under visible-light irradiation.

**Catalyst**	**k ± SD (min^{-1})**	**95% CI (min^{-1})**	**R^2**	**Tukey group**
**MgAlH**	0.00200 ± 0.00095	0.00081–0.00319	0.844	**d**
**2-CuMgH**	0.01500 ± 0.00129	0.01338–0.01662	0.994	**bc**
**3-CuMgH**	0.01700 ± 0.00084	0.01594–0.01806	0.998	**bc**
**4-CuMgH**	0.02400 ± 0.00273	0.02057–0.02743	0.994	**b**
**5-CuMgH**	**0.03200 ± 0.00549**	0.02510–0.03890	0.995	**a**
**6-CuMgH**	0.01900 ± 0.00124	0.01744–0.02056	0.998	**bc**

Values shown here are based on replicate-level regression estimates; different superscript letters indicate significant differences according to Tukey's HSD test at $p < 0.05$. **Reconstructed one-way ANOVA:** $F(5,12) \approx$ **42.43**, $p \approx$ **3.22×10^{-7}. Tukey HSD:** different letters indicate statistically significant differences at $p < 0.05$.

Table S7. Effect of initial solution pH on the photocatalytic removal efficiency of ciprofloxacin (CIP, 25 mg L^{-1}) over 5-CuMgH, with statistical comparison at 210 min.

Time (min)	Average CIP removal efficiency (%) at different initial pH values (%)					
	pH = 3.0	pH = 4.0	pH = 6.5 (original)	pH = 8.0	pH = 10.0	pH = 12.0
0	0	0	0	0	0	0
30_{ad}	4.5	5.5	5.6	5.6	4.6	3.0
60	30.1	61.7	56.1	67.9	40.9	6.5
90	75.7	82.4	88.1	82.2	63.0	14.1
120	86.2	88.9	92.2	89.1	69.9	13.7
150	87.7	92.1	94.4	90.8	71.4	14.5
180	91.5	93.7	95.1	90.9	74.3	15.7
210	92.6 ± 0.66^{b}	95.2 ± 0.26^{a}	95.5 ± 0.62^{a}	91.1 ± 0.26^{c}	78.1 ± 0.79^{d}	15.1 ± 0.44^{e}

Note: ^{ad} *Adsorption efficiency measured after 30 min of dark equilibration prior to light irradiation.* Values are presented as mean ± SD (n = 3). For statistical comparison, final CIP removal efficiencies after 210 min of visible-light irradiation were analyzed by one-way ANOVA followed by Tukey's HSD test. Different superscript lowercase letters within the 210 min row indicate statistically significant differences among the initial-pH conditions at $\alpha = 0.05$. One-way ANOVA at 210 min: $F(5,12) = 9997.59$, $p = 2.80 \times 10^{-21}$.

Table S8. Average ciprofloxacin (CIP) removal efficiencies achieved by 5-CuMgH at different initial CIP concentrations under visible-light irradiation.

Time (min)	Average CIP removal efficiency at different initial CIP concentrations (%)						
	10 ppm	20 ppm	30 ppm	40 ppm	50 ppm	75 ppm	100 ppm
0	0	0	0	0	0	0	0
30_{ad}	7.2	6.5	5.9	5.8	5.5	5.1	3.7
60	60.6	58.5	50.4	40.7	33.4	30.7	24.6
90	84.0	82.6	78.0	67.7	55.5	46.8	34.1
120	90.7	88.9	85.4	79.3	74.1	61.8	46.0
150	93.3	90.6	88.1	83.7	77.3	64.9	48.4
180	94.9	93.0	90.5	84.2	79.7	68.0	51.3
210	96.0	95.2	91.6	85.0	81.8	69.5	55.3

Note: ad: adsorption stage (30 min dark-equilibration period prior to visible-light irradiation).

Table S9. Comparison of CIP (25 mg L^{-1}) removal efficiencies under different experimental conditions: in the presence or absence of 5-CuMgH catalyst, under illumination or dark conditions, and with or without H_2O_2.

Time (min)	Average CIP (25 mg L^{-1}) removal efficiency (%)			
	5-CuMgH + Light, without H_2O_2	5-CuMgH + Light + H_2O_2	Without 5-CuMgH, with H_2O_2 and Light	5-CuMgH + H_2O_2, without Light
0	0	0	0	0
30_{ad}	6.1	5.6	0	6.2
60	4.6	56.1	6.0	44.5
90	5.7	88.1	6.7	64.9
120	7.7	94.2	7.6	76.7
150	8.4	94.4	7.6	81.4
180	9.3	95.1	8.3	88.1
210	9.4	95.5	8.5	89.9

Table S10. Regression-derived pseudo-first-order rate constants for CIP degradation under visible-light and dark conditions.

Experimental condition	*k* (min^{-1})	SD (min^{-1})	95% CI for *k* (min^{-1})	R^2	Tukey group
5-CuMgH + H_2O_2 + visible light	0.0320 ± 0.0010	0.0010	0.0032–0.0608	0.995	a
5-CuMgH + H_2O_2, without light	0.0150 ± 0.0005	0.0005	0.0127–0.0173	0.993	b

Note: *k* represents the pseudo-first-order rate constant obtained from linear regression of $\ln(C_0/C_t)$ versus irradiation time. The 95% CI represents the confidence interval of the fitted regression-derived *k*. SD represents the standard deviation among replicate estimates. Statistical comparisons were performed using one-way ANOVA followed by Tukey's HSD test. **One-way ANOVA:** $F(1, 4)$ = **771.28**, p = $\mathbf{1.00 \times 10^{-5}}$. **Tukey HSD:** Different lowercase letters indicate statistically significant differences between conditions at $p < 0.05$.

Table S11. Average removal efficiencies of 25 mg L⁻¹ ciprofloxacin (CIP) in the absence and presence of reactive-species scavengers.

Time (min)	Average CIP (25 mg L⁻¹) removal efficiency in the presence of different scavengers (%)				
	Without scavenger	Ascorbic acid 1 mM	Isopropyl alcohol 10 mM	Concentrated Isopropyl alcohol	KI 1 mM
0	0	0	0	0	0
30_{ad}	5.6	5.8	6.1	5.6	6.0
60	60.9	57.0	41.7	26.8	55.4
90	78.0	81.9	52.8	28.6	75.3
120	88.9	89.8	60.1	30.1	83.8
150	91.0	91.2	59.6	30.0	86.3
180	93.6	91.2	59.6	29.6	85.3
210	94.6	91.3	60.5	29.6	85.9

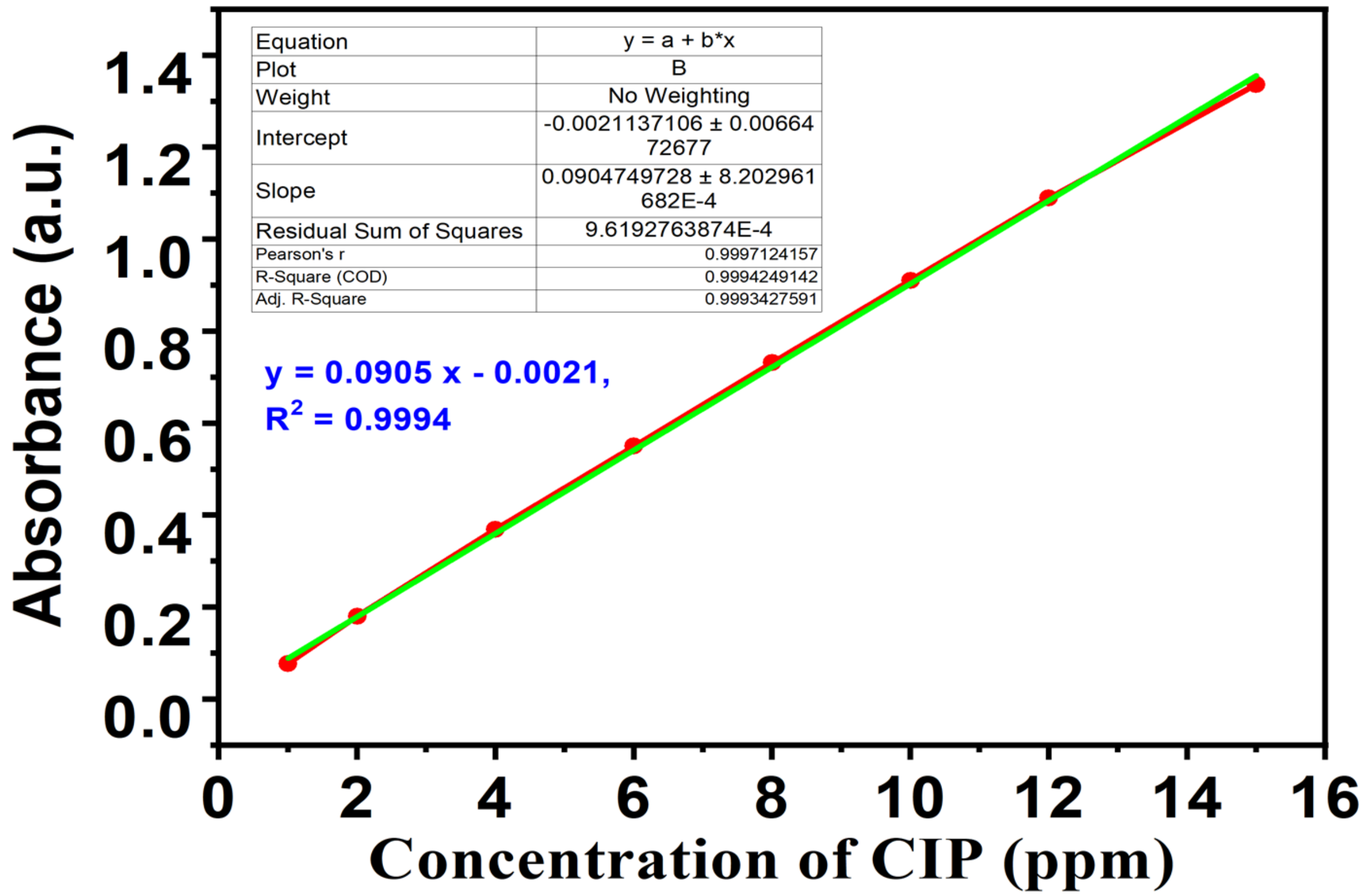

Fig. S1. Calibration curve showing the relationship between absorbance and ciprofloxacin (CIP) concentration at 272 nm.

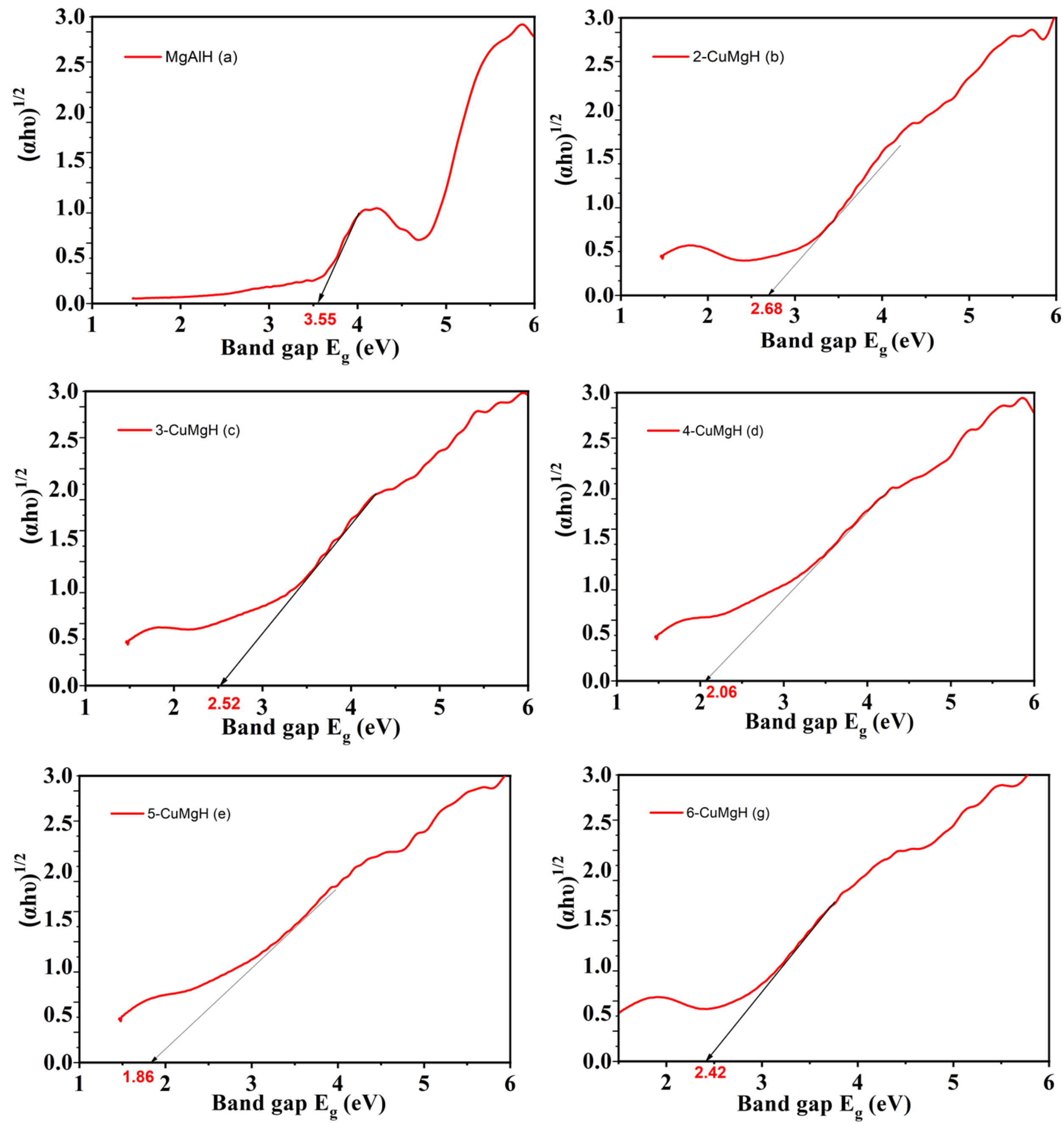

Fig. S2. Tauc plots used for determination of the optical band-gap energies (Eg) of MgAlH and Cu-modified MgAl hydrotalcites assuming indirect electronic transitions.

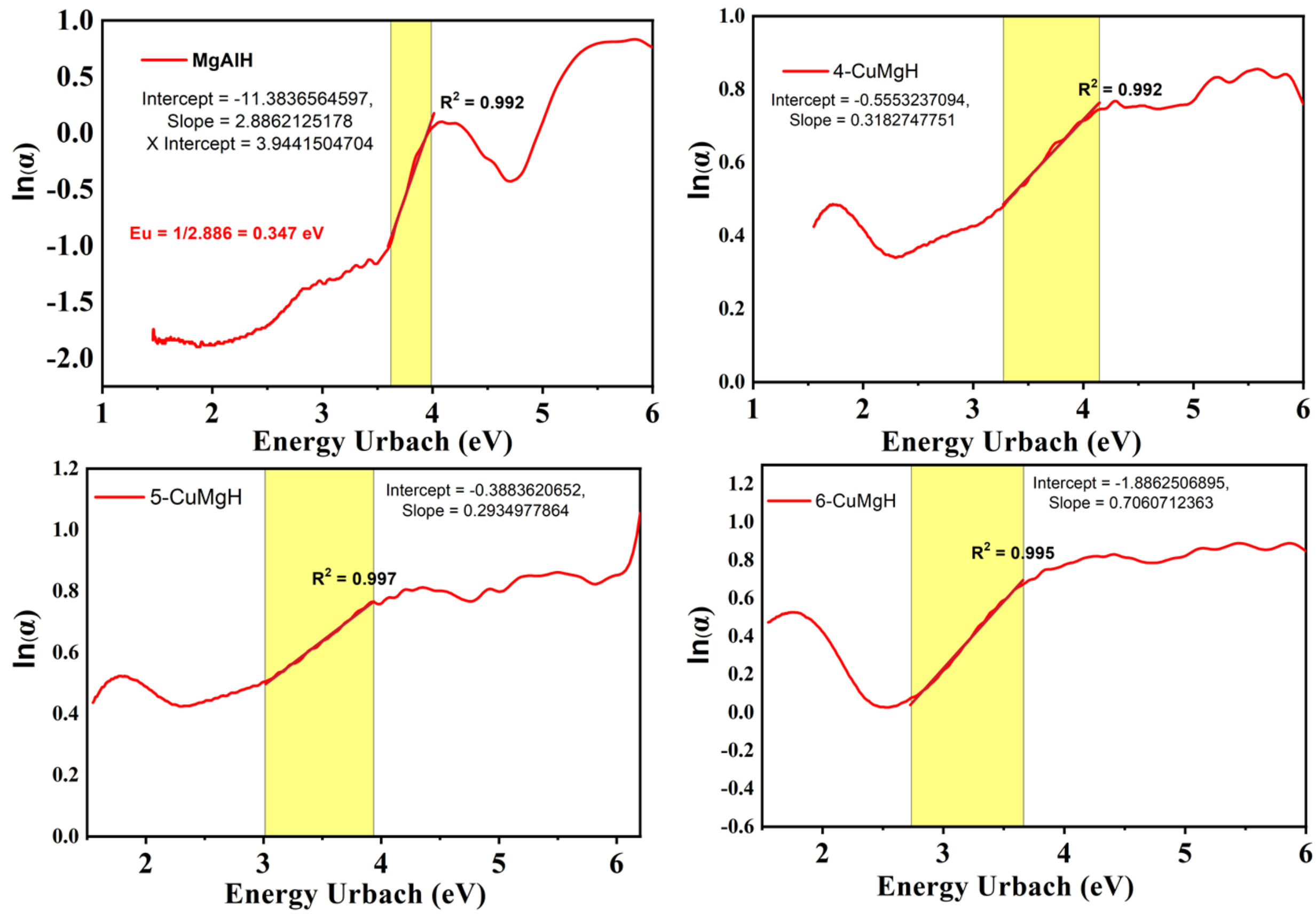

Fig. S3. Urbach plots used for determination of the Urbach energies (Eu) of MgAlH and Cu-modified MgAl hydrotalcite materials.

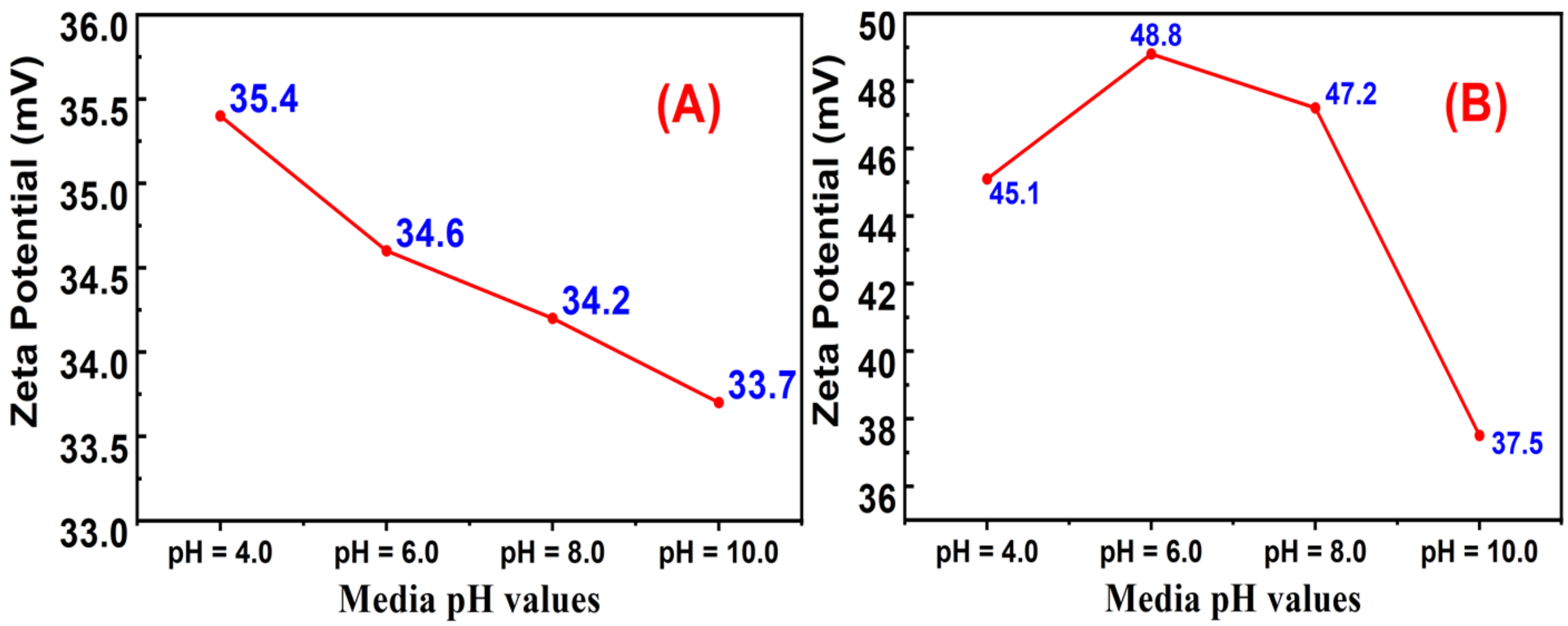

Fig. S4. pH-dependent zeta potentials of the representative materials MgAlH (A) and 5-CuMgH (B). These measurements were used to evaluate the possible contribution of surface charge and electrostatic interactions to ciprofloxacin adsorption.

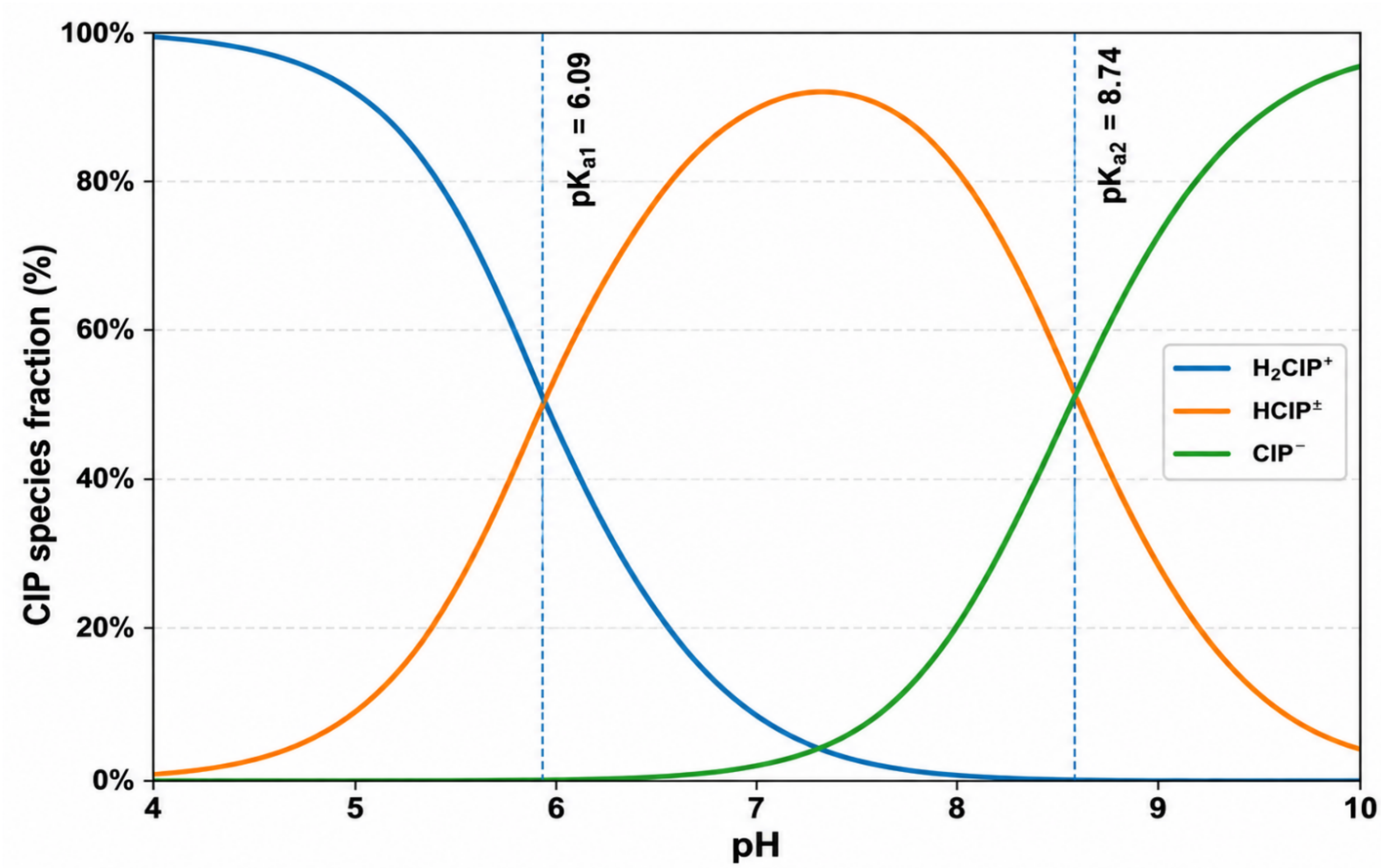

Fig. S5. Calculated pH-dependent speciation of ciprofloxacin (CIP) between pH 4 and 10 based on the reported pKa values of 6.09 and 8.74. The calculated distributions of H_2CIP^+, HCIP±, and CIP^- illustrate the progressive transition from cationic to zwitterionic and anionic forms with increasing pH.

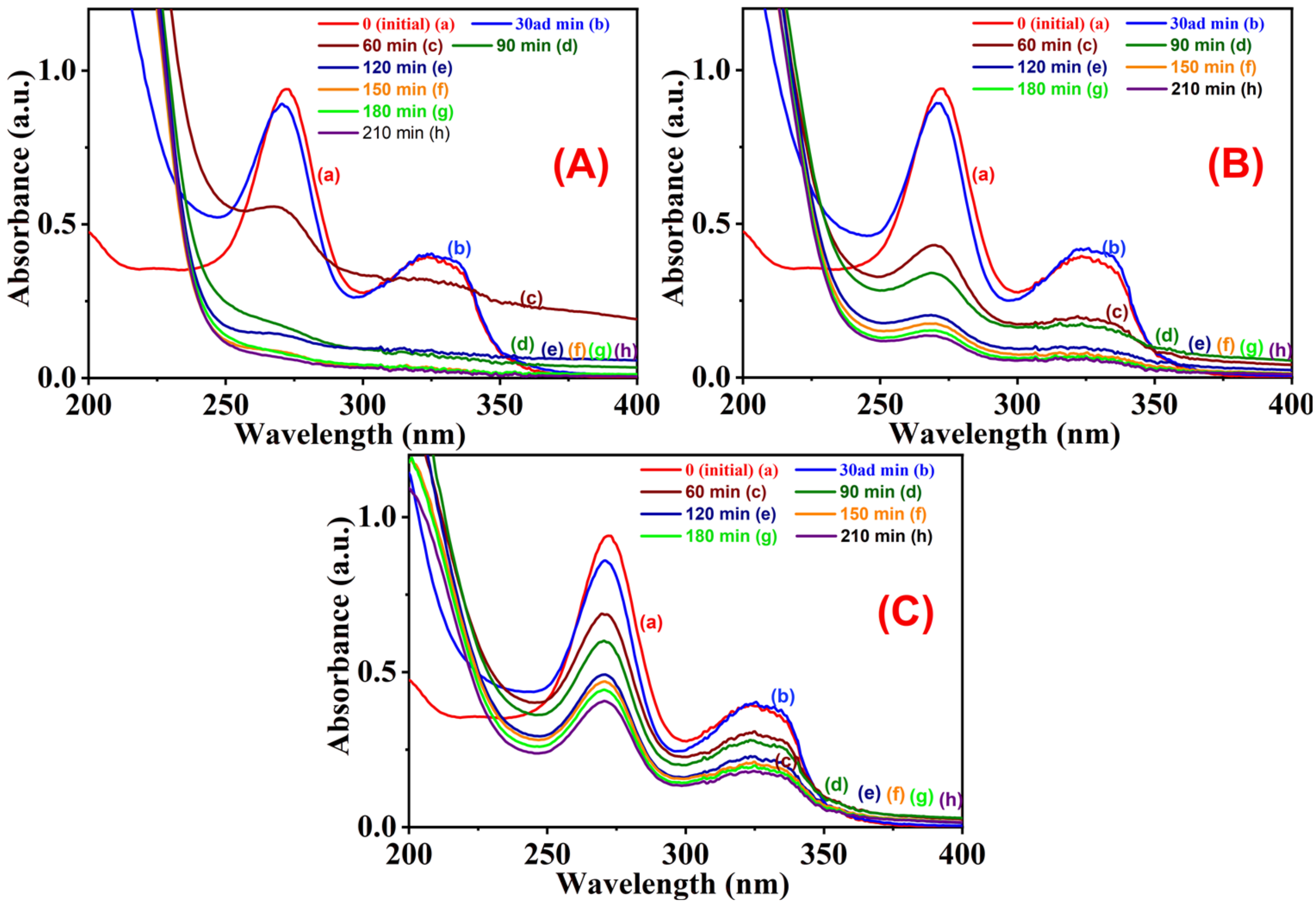

Fig. S6. UV–Vis spectral changes of ciprofloxacin (CIP) during photocatalytic degradation by 5-CuMgH at initial CIP concentrations of 10 mg L^{-1} (A), 50 mg L^{-1} (B), and 100 mg L^{-1} (C). Samples obtained from the 50 and 100 mg L^{-1} systems were diluted 5 and 10 times, respectively, before UV–Vis measurement to maintain the absorbance within the instrumental detection range.

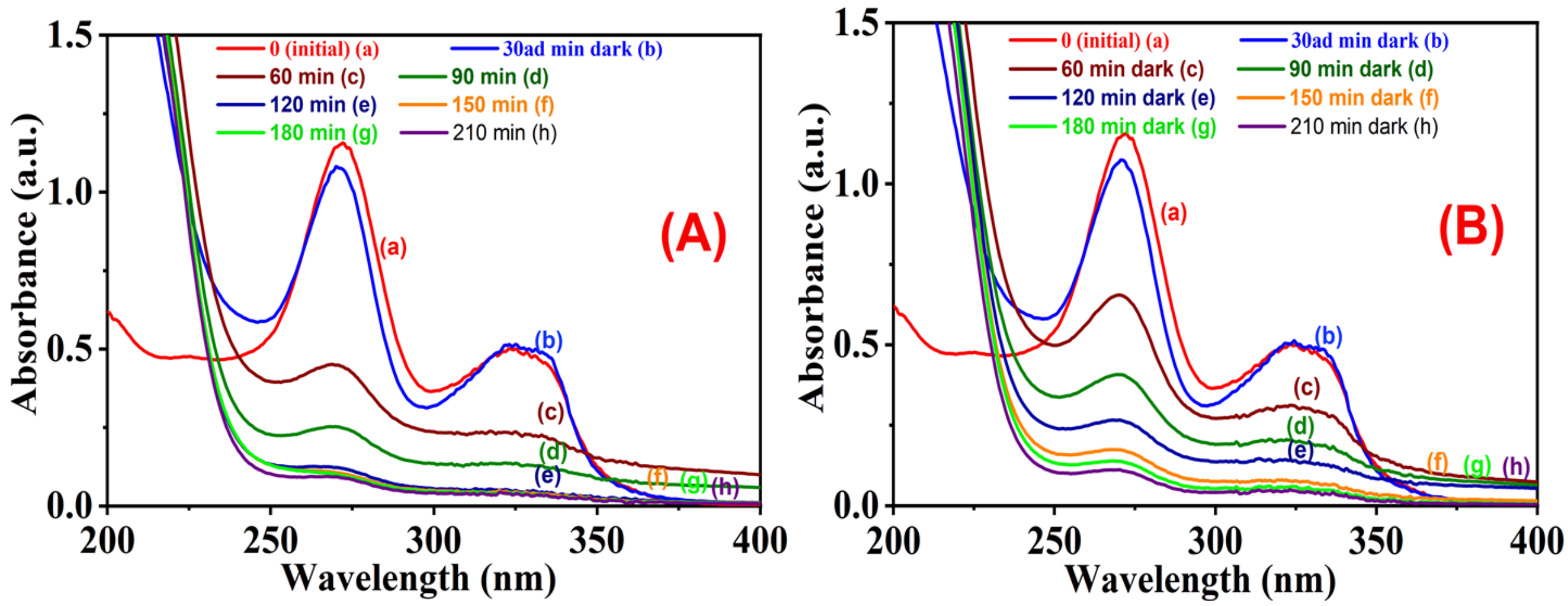

Fig. S7. UV–Vis spectral evolution of ciprofloxacin (CIP) during degradation over the 5-CuMgH/H_2O_2 system under visible-light irradiation (A) and dark conditions (B), illustrating the progressive disappearance of the characteristic CIP absorption band at approximately 272 nm.

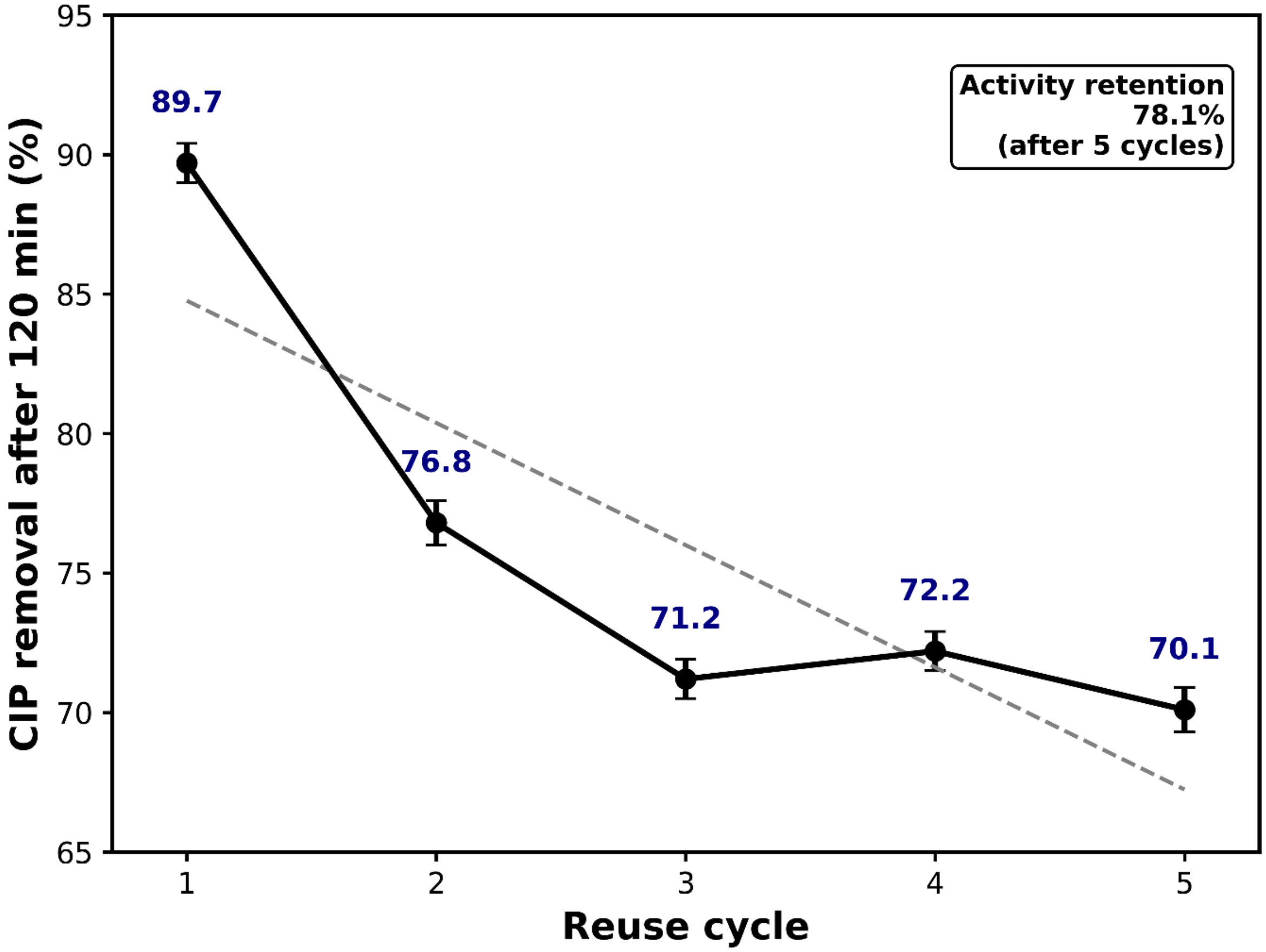

Fig. S8. Reusability performance of 4-CuMgH for ciprofloxacin removal after 120 min of visible-light irradiation over five consecutive cycles. Error bars represent standard deviations from three independent experiments. The photocatalytic activity remained at 78.1% of its initial value after the fifth reuse cycle.
